# Mitochondrial uncouplers inhibit clathrin-mediated endocytosis largely through cytoplasmic acidification

**DOI:** 10.1038/ncomms11710

**Published:** 2016-06-08

**Authors:** Wim Dejonghe, Sabine Kuenen, Evelien Mylle, Mina Vasileva, Olivier Keech, Corrado Viotti, Jef Swerts, Matyáš Fendrych, Fausto Andres Ortiz-Morea, Kiril Mishev, Simon Delang, Stefan Scholl, Xavier Zarza, Mareike Heilmann, Jiorgos Kourelis, Jaroslaw Kasprowicz, Le Son Long Nguyen, Andrzej Drozdzecki, Isabelle Van Houtte, Anna-Mária Szatmári, Mateusz Majda, Gary Baisa, Sebastian York Bednarek, Stéphanie Robert, Dominique Audenaert, Christa Testerink, Teun Munnik, Daniël Van Damme, Ingo Heilmann, Karin Schumacher, Johan Winne, Jiří Friml, Patrik Verstreken, Eugenia Russinova

**Affiliations:** 1 Department of Plant Systems Biology, VIB, 9052 Gent, Belgium; 2 Department of Plant Biotechnology and Bioinformatics, Ghent University, 9052 Gent, Belgium; 3 VIB Center for the Biology of Disease, Laboratory of Neuronal Communication, 3000 Leuven, Belgium; 4 Department for Human Genetics, and Leuven Institute for Neurodegenerative Diseases, KU Leuven, 3000 Leuven, Belgium; 5 Institute of Science and Technology Austria, 3400 Klosterneuburg, Austria; 6 Department of Plant Physiology, Umeå Plant Science Centre, Umeå University, 90187 Umeå, Sweden; 7 Department of Plant Physiology, Institute of Biochemistry and Biology, University of Potsdam, 14476 Potsdam, Germany; 8 Developmental Biology of Plants, Centre for Organismal Studies, Heidelberg University, 69120 Heidelberg, Germany; 9 Department of Plant Cell Biology, Swammerdam Institute for Life Sciences, University of Amsterdam, 1090 GE Amsterdam, The Netherlands; 10 Department of Cellular Biochemistry, Institute for Biochemistry and Biotechnology, Martin-Luther-University, 06120 Halle, Germany; 11 Compound Screening Facility, VIB, 9052 Ghent, Belgium; 12 Department of Forest Genetics and Plant Physiology, Umeå Plant Science Centre, Swedish University of Agricultural Sciences, 90183 Umeå, Sweden; 13 Department of Biochemistry, University of Wisconsin-Madison, Madison, Wisconsin 53706, USA; 14 Laboratory for Organic Synthesis, Department of Organic and Macromolecular Chemistry, Ghent University, 9000 Gent, Belgium

## Abstract

ATP production requires the establishment of an electrochemical proton gradient across the inner mitochondrial membrane. Mitochondrial uncouplers dissipate this proton gradient and disrupt numerous cellular processes, including vesicular trafficking, mainly through energy depletion. Here we show that Endosidin9 (ES9), a novel mitochondrial uncoupler, is a potent inhibitor of clathrin-mediated endocytosis (CME) in different systems and that ES9 induces inhibition of CME not because of its effect on cellular ATP, but rather due to its protonophore activity that leads to cytoplasm acidification. We show that the known tyrosine kinase inhibitor tyrphostinA23, which is routinely used to block CME, displays similar properties, thus questioning its use as a specific inhibitor of cargo recognition by the AP-2 adaptor complex via tyrosine motif-based endocytosis signals. Furthermore, we show that cytoplasm acidification dramatically affects the dynamics and recruitment of clathrin and associated adaptors, and leads to reduction of phosphatidylinositol 4,5-biphosphate from the plasma membrane.

## Abstract (web summary)

Plant cells maintain strict proton gradients over different membranes. Here, Dejonghe *et al*. show that several protonophores, including the known tyrosine kinase inhibitor TyrphostinA23, inhibit clathrin-mediated endocytosis by disturbing these gradients and causing cytoplasmic acidification.

## Graphical abstract

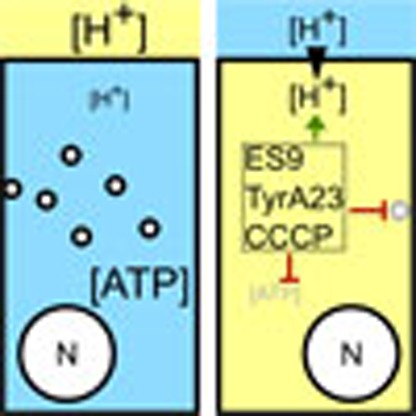

Clathrin-mediated endocytosis (CME) is a major pathway for the uptake of extracellular and plasma membrane material in all eukaryotic cells^1^. CME is critical for the ability of cells to respond to environmental changes, including pathogen entry, synaptic vesicle turn-over, and the constitutive or regulated internalization of membrane-bound receptors and their ligands, which in turn might influence signalling outputs^1^. In plant cells, CME depends on an evolutionarily conserved core machinery that, in addition to clathrin, comprises the adaptor protein complex-2 (AP-2) and dynamins as well as the newly discovered TPLATE adaptor complex (TPC)^2^,^3^. Mainly classical genetic approaches have contributed to our current understanding about the mechanisms of CME in yeast, metazoans, and plants^1^,^2^,^3^. In addition, chemical genetics also has the potential to facilitate studies of CME by providing small molecule effectors that can interfere with CME in a conditional manner^4^. An example of such a CME inhibitor is tyrphostinA23 (TyrA23). TyrA23 is a tyrosine-like small molecule originally developed as a substrate-competitive inhibitor of mammalian tyrosine kinases^5^. Subsequently, TyrA23 was found to inhibit CME, presumably through its ability to interfere with the interaction between the tyrosine-based internalization motifs present in different endocytic cargos and the medium subunit of the clathrin-associated adaptor complex AP-2 (refs 6, 7). TyrA23 was mainly exploited as a CME inhibitor in plant cells by many researchers, including us (Supplementary Data 1), despite the fact that its mode of action has never been well characterized in this system. Recent studies have shown that TyrA23 inhibits flagellin 22 (flg22)-elicited reactive oxygen species formation^8^, indicating that TyrA23, affects not only CME but also other biochemical and/or cellular processes. Besides TyrA23, other chemical tools have been utilized to study the endocytosis in mammalian and yeast systems, such as the dynamin inhibitor dynasore^9^ and the pitstops^10^ that target the clathrin terminal domain, but these small molecules also display off-target effects, including inhibition of clathrin-independent endocytosis^11^,^12^,^13^,^14^. Although dynasore has been used as a CME inhibitor in plants^15^, reports on the activity of other CME inhibitory compounds in plant cells are still lacking.

Here, we identified and characterized Endosidin9 (ES9), a small molecule inhibitor of CME in *Arabidopsis thaliana* and *Drosophila melanogaster*. We reveal that this molecule uncouples mitochondrial oxidative phosphorylation, a mode of action that is shared with TyrA23. We demonstrate that the effects of ES9 and TyrA23 on CME are not dependent on mitochondrial dysfunction and ATP depletion, but rather on their uncoupling activity at other membranes, leading to acidification of the cytoplasm. Acidification, but not plasma membrane depolarization, caused a dramatic increase in the lifetimes of clathrin and associated adaptors and led to a reduction of the phosphatidylinositol 4,5-biphosphate (PI(4,5)P_2_), thereby most likely inhibiting clathrin-coated pits formation.

## Results

### ES9 blocks CME in different systems

**Figure 1:**
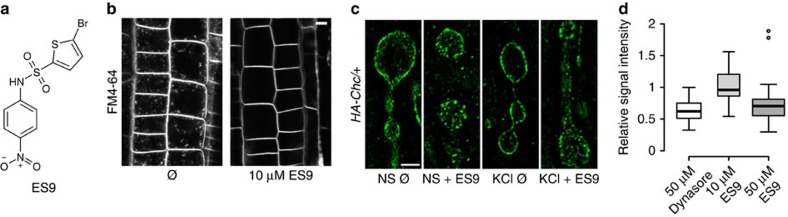
ES9 inhibits CME in different systems. (**a**) Chemical structure of ES9. (**b**) Strongly reduced FM4-64 uptake (2 μM, 30 min) in *Arabidopsis* root cells in the presence of ES9 (10 μM) when compared with mock treatment (DMSO, Ø). (**c**) Super resolution immunofluorescence images of boutons of the neuromuscular junction (NMJ) in third instar *Drosophila* larvae positive for HA-tagged clathrin heavy chain (HA-CHC) comparing mock with 10 μM ES9 (30 min), in both non-stimulated (NS) or 90 mM KCl-stimulated NMJs to induce uptake. (**d**) Boxplot representation of transferrin uptake in HeLa cells after 20 min in the presence of 50 μM dynasore and 10 or 50 μM ES9. The signal intensity is plotted relative to mock (DMSO) and shows a concentration-dependent inhibition of uptake by ES9. Center lines show the medians; box limits indicate the 25th and 75th percentiles as determined by R software; whiskers extend 1.5 times the interquartile range from the 25th and 75th percentiles; outliers are represented by dots. The box width is relative to sample size (*n*=35, 29, and 61 cells). Scale bars, 5 μm in **b**; 2 μm in **c**.

To identify chemical inhibitors of CME, we analysed a set of 123 small molecules that had previously been selected as endomembrane trafficking modifiers in a screen for inhibitors of pollen germination and pollen tube growth^16^ of tobacco (*Nicotiana tabacum*). From this set of small molecules we identified ES9 (Fig. 1a) based on its ability to inhibit the uptake of the lipophilic styryl endocytic tracer dye *N*-(3-triethylammoniumpropyl)-4-(6-(4-(diethylamino)phenyl)hexatrienyl)pyridinium dibromide (FM4-64)^17^ in *Arabidopsis* root epidermal cells with half-maximal inhibitory concentration (IC_50_) of 5 μM (Fig. 1b; Supplementary Fig. 1a). To rule out that the ES9 effect was not limited to FM4-64, we tested whether ES9 blocked the uptake of the fluorescently labelled Alexa fluor 674 castasterone (AFCS) analogue that binds the brassinosteroid receptor and undergoes CME^18^. In the absence of ES9, AFCS (20 μM, 30 min pulse, 20 min chase) was found to traffic to the vacuole in *Arabidopsis* root cells, but in the presence of ES9, AFCS was not internalized, indicating that its effect was not limited to FM dyes (Supplementary Fig. 1b).

To examine whether ES9 had the potential to act as a general inhibitor of CME, we assessed if ES9 interfered with the synaptic vesicle formation in *Drosophila* neurons and with the uptake of transferrin in HeLa cells, two clathrin-dependent processes^1^,^19^,^20^. As a model synapse we used the *Drosophila* third instar neuromuscular junction (NMJ), where nerves were stimulated for 5 min with 90 mM KCl to enhance endocytosis and exocytosis^19^,^20^ in the presence of FM1-43, a dye that becomes internalized in newly formed vesicles upon nerve stimulation. Treatment with ES9 (10 μM, 30 min) did not block the uptake of FM1-43, but induced its accumulation into large membranous structures (Supplementary Fig. 1c). Transmission electron microscopy (TEM) studies confirmed the formation of abnormal membrane inclusions and the lack of normal-sized synaptic vesicles after ES9 application (Supplementary Fig. 1d), a phenotype reminiscent of acute loss of clathrin heavy chain (CHC)^19^, or dynamin^20^ functions. To support this observation, we assessed the localization of the CME machinery before or after stimulation of NMJs in the presence of ES9 (10 μM, 30 min). Visualization of CHC (Fig. 1c) and the plasma membrane clathrin-associated AP-2 complex (Supplementary Fig. 1e) by super resolution immunofluorescence revealed that, after nerve stimulation, ES9 prevented their recruitment from the bouton centre to the periphery, where vesicles are endocytosed, a phenotype also observed when either dynamin or CHC were impaired^19^,^20^. Similarly, the localization, but not the cellular levels of PI(4,5)P_2_, was affected following ES9 treatment (Supplementary Fig. 1f,g). Finally, ES9 inhibited the uptake of transferrin in HeLa cells, a hallmark CME tracer^1^, to an extent comparable to that of the known inhibitor dynasore^9^ when used at a high concentration (50 μM, 20 min) (Fig. 1d). Altogether these data show that ES9 was able to affect clathrin-dependent processes in different model systems.

### ES9 arrests the dynamics of CME and membrane trafficking

**Figure 2:**
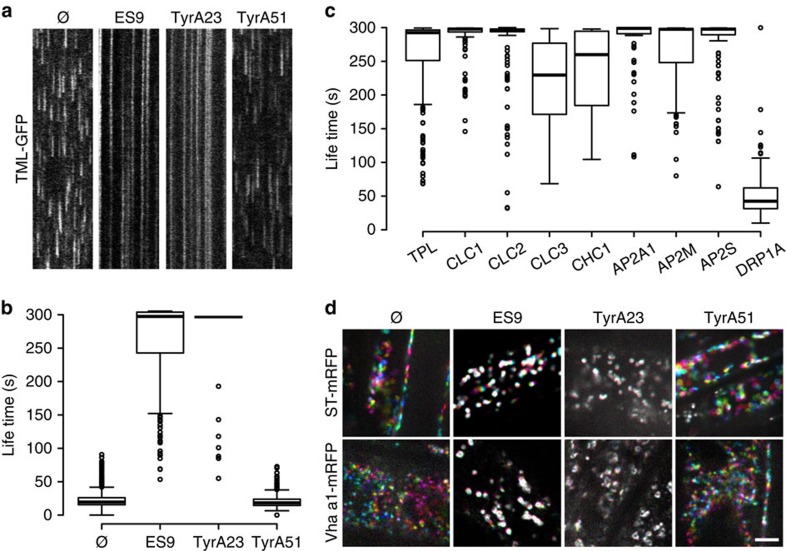
ES9 inhibits CME dynamics and organelle movement in the cytoplasm. (**a**) Kymographs representing a line trace (horizontal axis) over a time period (vertical axis, 5 min), taken from the respective spinning disc movies (2 f s^−1^) of *Arabidopsis* root cells and illustrating the life times of endocytic spots labelled by TML-GFP for the control treatment (DMSO, Ø), 10 μM ES9, 50 μM tyrphostinA23 (TyrA23), and 50 μM TyrA51. (**b**) Boxplot representation of measured endocytic spot life times. *n*=1646, 171, 121, and 868 measurements. (**c**) Boxplot representation of kymograph-based life time of foci at the plasma membrane in the presence of 10 μM ES9 for green fluorescent protein (GFP)-tagged TPLATE, clathrin light chain (CLC), clathrin heavy chain (CHC), the adaptor protein complex-2 (AP-2), represented by the AP2A1, AP2M, and AP2S subunits, and the dynamin DRP1A. *n*=204, 125, 142, 243, 60, 150, 176, 121, and 247 measurements. (**d**) Visualization of the Golgi (ST-mRFP) and the *trans*-Golgi network (TGN, VHA-a1-mRFP) in *Arabidopsis* root cells treated with 10 μM ES9, 50 μM TyrA23, 50 μM TyrA51, and mock (DMSO, Ø) (5 min). Images are composed of six differentially coloured images taken with a 10-s interval. Movement is illustrated by the presence of the different colours, whereas white indicates static compartments. Boxplot centre lines show the medians; box limits indicate the 25th and 75th percentiles as determined by R software; whiskers extend 1.5 times the interquartile range from the 25th and 75th percentiles; outliers are represented by dots. Scale bar, 5 μm.

As ES9 inhibited several clathrin-dependent processes, we tested its effect on the dynamic recruitment of the CME machinery. The dynamic behaviour of TPLATE muniscin-like fused to green fluorescent protein (GFP) (TML-GFP), a subunit of the CME adaptor TPC^3^, was evaluated in *Arabidopsis* root cells after ES9 treatment and compared with the effect of the known CME inhibitor TyrA23 (Supplementary Data 1). An approximate average dwell time of 20 s was observed for TML-GFP in the plasma membrane in control conditions, including treatments with either dimethyl sulfoxide (DMSO) or the inactive TyrA23 analogue TyrA51 (50 μM; Fig. 2a,b; Supplementary Data 1). However, for seedlings treated with 10 μM ES9 or 50 μM TyrA23, the TML-GFP-labelled foci at the plasma membrane appeared stationary, often with a lifetime exceeding several minutes (Fig. 2a,b). The loss of dynamic behaviour occurred rapidly, as images were acquired 3–5 min after the start of the treatments. ES9 also inhibited the dynamic behaviour of another TPC subunit, TPLATE-GFP^3^, the AP-2 complex subunits AP2A1-GFP^21^, AP2M-GFP^22^, and AP2S-GFP^23^, and clathrin represented by clathrin light chain 1 (CLC1)-GFP, CLC2-GFP, CLC3-GFP and CHC1-GFP, but notably did not impair recruitment and only slightly increased the lifetime of the red fluorescent protein (RFP)-dynamin-related protein1A (DRP1A)^24^ at the plasma membrane (Fig. 2c; Supplementary Fig. 3a).

ES9 also affected the PI(4,5)P_2_, component involved in CME, which was visualized with the 2xPH^PLC^ (P24Y) marker^25^. Following application with ES9 (10 μM, 30 min) P24Y localized mainly to the cytoplasm, in contrast to its predominant plasma membrane localization under control conditions, whereas phosphatidylinositol 4-phosphate (PI4P) maintained its plasma membrane localization, as illustrated by the 2xPH^FAPP1^ (P21Y)^25^ marker (Supplementary Fig. 2a,e). The reduced plasma membrane recruitment of the PI(4,5)P_2_ biosensor was correlated with a decrease in PI(4,5)P_2_ levels after ES9 treatment (Supplementary Fig. 2b,c).

We next tested whether treatments with ES9 and TyrA23 would compromise the motility of Golgi and *trans*-Golgi network (TGN)/early endosome (EE) compartments, and the actin-cytoskeleton dynamics. When seedlings expressing Golgi (ST-mRFP)^26^ and TGN/EE (VHA-a1-mRFP)^27^ markers were treated with ES9 (10 μM) or TyrA23 (50 μM), their dynamic behaviour was compromised after 3–10 min when compared with the control treatments with either TyrA51 (50 μM) or DMSO (Fig. 2d). Inhibition of the dynamic behaviour probably explains the observation that co-application of ES9 or TyrA23 with Brefeldin A (BFA), an inhibitor of the BFA-sensitive ADP-ribosylation factors guanine nucleotide exchange factors (ARF-GEFs)^28^, prevented the formation of the BFA body (Supplementary Fig. 3b). Moreover, 10 μM ES9 treatment inhibited actin-cytoskeleton dynamics after 10 min, as visualized by the actin binding domain (ABD)-GFP^29^ (Supplementary Fig. 3c). These results indicate that ES9 and TyrA23 affected the dynamics and recruitment of essential CME components at the plasma membrane, thereby hindering endocytosis, as well as affecting Golgi, TGN/EE, and actin dynamics, reflected in the inhibition of BFA body formation.

### ES9 affects energy production

**Figure 3:**
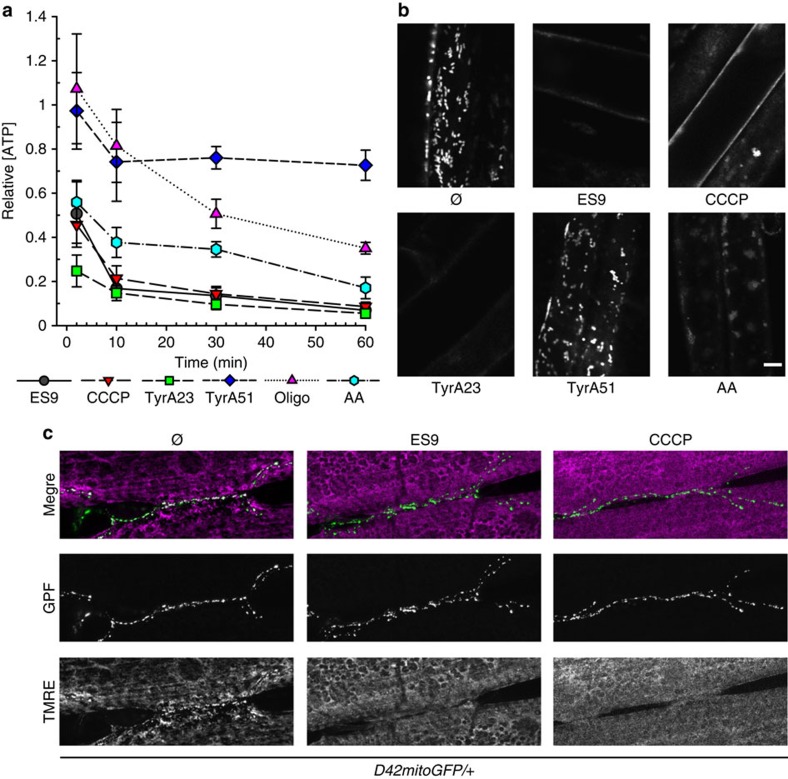
ES9 is a mitochondrial uncoupler. (**a**) ATP measurements in the presence of 10 μM ES9, 1 μM carbonyl cyanide m-chlorophenyl hydrazine (CCCP), 50 μM tyrphostinA23 (TyrA23), 50 μM TyrA51, 20 μM antimycin A (AA), and 10 μM oligomycin (Oligo), showing various degrees of ATP depletion relative to mock treatment. Results were combined from three independent experiments. Error bars, s.e.m. (**b**) Confocal images of labelled mitochondria in *Arabidopsis* root cells (250 nM MitoTracker Red CM-H2XRos) in the presence of 10 μM ES9, 1 μM CCCP, 50 μM TyrA23, 50 μM TyrA51, 20 μM AA, and the mock (DMSO, Ø) for 30 min. The absence of mitochondrial staining implies that the electron transport is uncoupled or inhibited. (**c**) Tetramethylrhodamine ethyl ester (TMRE) labelling of D42mitoGFP positive mitochondria in *Drosophila* neuromuscular junctions compared to mock (1% DMSO (v/v) in HL-3 medium, 15 min) with 10 μM ES9 (30 min pre-treatment, 15 min treatment) and 10 μM CCCP (15 min). Reduced TMRE staining points to disruption of the mitochondrial function and reveal more depolarized mitochondria. Scale bars, 5 μm.

As CME is an energy-dependent process^1^ and ATP depletion has been shown to severely reduce CME in mammalian cells^30^,^31^, the capacity of ES9 to affect cellular ATP was assessed in dark-grown *Arabidopsis* PSB-D cell cultures. The ATP production inhibitors, antimycin A (AA)^32^ and oligomycin^33^, which interfere with mitochondrial function, and the protonophore, carbonyl cyanide m-chlorophenyl hydrazone (CCCP)^34^ were included as positive controls, whereas TyrA23 and TyrA51 were used as putative negative controls. ES9 (10 μM) induced a rapid initial depletion of ATP to ∼50% cellular ATP after 2 min treatment. The ATP concentration decreased further, reaching a base line of ∼10% remaining ATP when compared with mock treatment (Fig. 3a). Interestingly, 50 μM TyrA23, but not TyrA51, was also found to induce a reduction in the cellular ATP, in this case of ∼75% after 2 min, subsequently stabilizing at ∼10% residual ATP when compared with the mock control (Fig. 3a). The trends of ATP depletion profiles of ES9 and TyrA23 were similar to those of AA (20 μM) and CCCP (1 μM), but differed from that of oligomycin (10 μM) (Fig. 3a), of which the ATP content decrease occurred more gradually. To establish whether the observed ATP depletion resulted from acute cytotoxic effects of the compounds, we assessed the viability of PSB-D cells treated with the various compounds by staining with the viability tracer fluorescein diacetate (FDA)^35^. In all cases, the FDA profiles increased similarly to the control treatment (Supplementary Fig. 4a). These results suggest that the impact of ES9 and TyrA23 on ATP production was not due to an acute loss in cell viability.

As PSB-D cell cultures were grown in the dark, the mitochondria-derived ATP represented the main cellular ATP source. To test the hypothesis that ES9 and TyrA23 alter mitochondrial functions, we isolated mitochondria from *Arabidopsis* leaves and performed a series of respiratory measurements *ex cellulo*. Mock treatment did not affect the mitochondrial electron transport chain (mETC), because high respiratory control ratios were maintained (Supplementary Fig. 4b). Addition of CCCP (1 μM) led to a characteristically rapid and transient increase in the respiratory rate as well as to a clear uncoupling between oxidative and phosphorylation reactions within the mETC, as shown by the respiratory control ratio drop in spite of an ADP addition. Even at low concentrations, ES9 (1 μM) and TyrA23 (5 μM), but not TyrA51 (5 μM), had an effect similar to that of CCCP on respiratory properties (Supplementary Fig. 4c). The dissipation of the mitochondrial membrane potential in the presence of ES9 and TyrA23 was confirmed *in vivo* in cells of the early differentiation zone of *Arabidopsis* roots with the MitoTracker Red CM-H2XRos dye that stains mitochondria in live cells depending on their membrane potential^36^. The mitochondrial labelling was not affected after mock (DMSO) and TyrA51 treatments (50 μM, 30 min) (Fig. 3b), whereas in cells treated for the same time with CCCP (1 μM), ES9 (10 μM), AA (20 μM), or TyrA23 (50 μM) labelling of mitochondria was not detected (Fig. 3b), implying that the electron transport was uncoupled or inhibited.

The ability of ES9 to deplete ATP was also evaluated in *Drosophila* and in human Jurkat cells (Supplementary Fig. 4d,e). Similar to *Arabidopsis*, ES9 (10 μM) depleted ATP in *Drosophila* S2 cell cultures like CCCP (10 μM), TyrA23 (50 μM), and AA (50 μM), as well as the otherwise considered inactive analogue TyrA51 (50 μM) (Supplementary Fig. 4d). ATP was also depleted by ES9 (50 μM, 3 h) in Jurkat cells grown in the presence of glucose or galactose to account for the Crabtree effect^37^ (Supplementary Fig. 4e). Notably, 50 μM of the known dynamin inhibitor dynasore^9^ did not affect ATP concentrations, whereas TyrA23 and TyrA51 at 350 μM had the strongest effect in this system. In agreement, TEM studies revealed swollen mitochondria in synaptic boutons in *Drosophila* after ES9 treatment (10 μM, 30 min) (Supplementary Fig. 1d) and neuronal mitochondria labelling with tetramethylrhodamine ethyl ester (TMRE)^38^ was greatly impaired in the presence of ES9 (10 μM, 30 min) and CCCP (10 μM, 30 min), confirming disruption of the mitochondrial function (Fig. 3c; Supplementary Fig. 4f). Taken together, the above results show that ES9, as well as TyrA23, are potent mitochondrial uncouplers in different systems.

### ATP depletion does not immediately block CME

In metazoans, AA, oligomycin, and mitochondrial depolarization with CCCP have been linked to CME inhibition mainly through a metabolic block^30^,^39^,^40^. In agreement, a reduction in transferrin internalization in HeLa cells (Supplementary Fig. 5a) and in FM1-43 uptake in stimulated boutons in *Drosophila* (Supplementary Fig. 5b,c) was observed after CCCP and TyrA23 applications. To investigate whether the ES9- and TyrA23-induced CME inhibition was triggered by ATP depletion, we evaluated the effect of CCCP, AA, and oligomycin on endocytosis in *Arabidopsis*, given that the CME inhibitors ES9 and TyrA23 also depleted cellular ATP. Similar to ES9 and TyrA23, CCCP (1 μM, 30 min) induced a complete inhibition of FM4-64 uptake with an IC_50_ of 0.61 μM, compared with a IC_50_ of 36 μM for TyrA23 (Supplementary Fig. 5d–g). In contrast, the inhibition of FM4-64 uptake, as measured from the plasma membrane/cytoplasmic signal intensity ratio, was only slightly affected when FM4-64 was co-applied with AA (50 μM, 30 min) or added after pre-treatment with oligomycin (50 μM, 30 min) and imaged after an additional 30 min (Supplementary Fig. 5d,e). In cells pre-treated with oligomycin, the ATP levels were similar to those treated with AA, CCCP and ES9 (Fig. 3a), indicating that ATP depletion alone is not sufficient to completely block FM4-64 uptake in plant cells. Consistently, AA treatment increased the lifetime of the TML-labelled foci in the plasma membrane, but did not block their dynamics, whereas the effect of CCCP resembled that of ES9 and TyrA23 (Fig. 2a,b; Supplementary Fig. 5h,i). Similar to the effects of ES9 and TyrA23, treatment with CCCP (1 μM) also compromised the movement of the TGN/EE and blocked BFA body formation (Fig. 2d; Supplementary Fig. 3b,d). However, when seedlings expressing the Golgi (ST-mRFP) or the TGN/EE (VHA-a1-mRFP) markers were treated with AA (20 μM), TGN/EE showed a dynamic behaviour comparable to that of the control, whereas the Golgi moved slower than the mock-treated sample (Supplementary Fig. 3d). In summary, these results show that although ATP depletion significantly reduced the dynamics of the CME machinery and the Golgi and TGN/EE movements in *Arabidopsis* cells, it was not the primary cause of the observed fast CME inhibition by ES9.

### Cytoplasmic acidification inhibits CME in *Arabidopsis*

**Figure 4:**
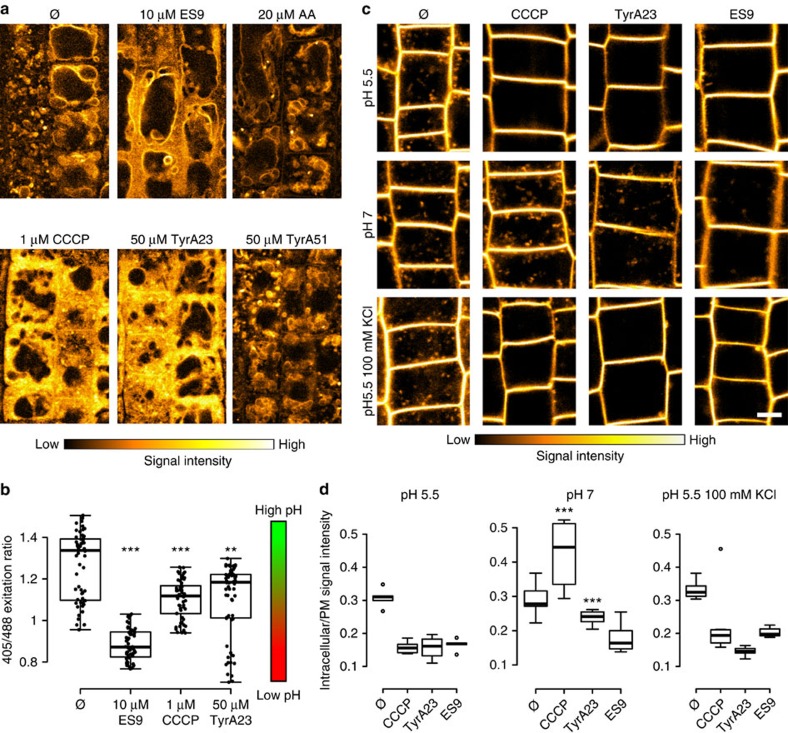
Cytoplasmic acidification inhibits CME. (**a**) Confocal images of *Arabidopsis* root cells stained with Lyso Tracker Red DND 99 (30 min) followed by an additional 30 min in the presence of DMSO (Ø), ES9, antimycin A (AA), carbonyl cyanide m-chlorophenyl hydrazine (CCCP), tyrphostinA23 (TyrA23), and TyrA51. The presence of ES9, CCCP and TyrA23 relocalized the dye from the tonoplast to the cytoplasm (**b**) Boxplot representation of cytoplasmic pH measurements using pH-GFP in the presence of 10 μM ES9, 50 μM TyrA23, and 1 μM CCCP, showing cytoplasmic acidification after treatment. *n*=59, 60, 54, and 61 seedlings respectively. Individual data points are represented. (**c**) Confocal images comparing FM4-64 uptake (30 min) at pH 5.5, pH 7, or pH 5.5 and 100 mM KCl in the presence of mock (DMSO, Ø), 1 μM CCCP, 50 μM TyrA23, and 10 μM ES9. (**d**) Quantification of the FM4-64 signal intensity under the different conditions in **c**. Values represent the ratio of cytoplasmic/plasma membrane signal intensity. Stars indicate significance compared to the corresponding treatment at pH 5.5. **P<0.01 and ***P<0.001 with a Kruskal-Wallis analysis of variance (ANOVA) on Ranks. The boxplot centre lines show the medians; box limits indicate the 25th and 75th percentiles as determined by R software; whiskers extend 1.5 times the interquartile range from the 25th and 75th percentiles; outliers are represented by dots. For each condition five to seven seedlings were measured. Scale bars, 5 μm.

The difference in phenotypes between AA and oligomycin relative to CCCP, TyrA23, and ES9 led us to hypothesize that TyrA23 and ES9 might act similarly to CCCP as general protonophores, thereby uncoupling proton gradients throughout the entire cell and resulting in the acidification of the cytoplasm^41^. To test this hypothesis, *Arabidopsis* seedlings were pre-incubated with Lyso Tracker Red DND 99, a dye that labels acidic compartments^42^ for 30 min before treatment with the various compounds. Mock treatment (DMSO) (Supplementary Fig. 5j) and treatments with 50 μM TyrA51 or 20 μM AA for 30 min had no effect on the Lyso Tracker Red DND 99 staining relative to untreated cells (Fig. 4a). However, addition of CCCP (1 μM), ES9 (10 μM) or TyrA23 (50 μM) for 30 min resulted in a substantial increase of cytoplasmic Lyso Tracker Red DND 99 labelling (Fig. 4a), suggesting a change in proton concentration throughout the cell, possibly as a consequence of proton gradient dissipation over the tonoplast and the plasma membrane. Therefore, we measured the pH in the vacuole and the cytoplasm of root cells in the presence of the small molecules. By using a pH-GFP cytoplasmic marker^43^, a significant acidification of the cytoplasm was observed for 10 μM ES9, 1 μM CCCP, and 50 μM TyrA23 (Fig. 4b), whereas the vacuolar pH was less affected in the presence of the same small molecules and considerably less affected than the positive controls, such as concanamycinA (ConcA) treatment and the genetic removal of the V-type proton pump in the *vha-a2vha-a3* double mutant^44^ (Supplementary Fig. 5k). Given that the vacuolar pH showed only a modest increase, we hypothesized that cytoplasmic acidification could be attributed to the influx of extracellular protons from the acidic apoplast across the plasma membrane, where the main uncoupling activity of the compounds might occur. A prediction based on this model is that acidification of the cytoplasm and inhibition of CME would be prevented by increasing the pH of the apoplast. Therefore, FM4-64 uptake in the presence of the various compounds was evaluated in seedlings incubated in alkaline growth medium (pH 7). Increasing the pH of the incubation medium from 5.5 to 7 had no effect on FM4-64 internalization (30 min) (Fig. 4c,d). In contrast, the ability of CCCP (1 μM, 30 min) or TyrA23 (50 μM, 30 min) to block FM4-64 uptake into cells was inhibited in seedlings incubated in media with a pH >6.5 (Fig. 4c,d; Supplementary Fig. 6g). Intriguingly ES9 treatment (10 μM, 30 min) at pH 7 remained sufficient to inhibit FM4-64 uptake (Fig. 4c,d), suggesting that besides acidification, ES9 probably has additional effects on the process of CME.

As protonophores induce movement of protons across the cell membranes, they also cause a change in the plasma membrane potential^41^. We next addressed the question of whether plasma membrane depolarization caused by the protonophores impaired CME. For this experiment the membrane potential was dissipated by applying potassium ions (K^+^) or valinomycin (Val)^45^, an ionophore that binds K^+^ and facilitates their transfer across lipid bilayers. To monitor the plasma membrane potential we used the voltage-sensitive fluorescent dye bis-(1,3-dibutylbarbituric acid)-trimethine oxonol (DiBAC4(3))^46^. When bound to depolarized membranes this dye exhibits an enhanced fluorescence and conversely hyperpolarization is indicated by a decrease in fluorescence. Medium supplemented with 100 mM KCl was effective in depolarizing the plasma membrane in the epidermis of *Arabidopsis* roots as detected by DiBAC4(3) staining (Supplementary Fig. 6a) without causing cytoplasmic acidification, as estimated by the pH-GFP marker^43^ and the Lyso Tracker Red DND 99 (Supplementary Fig. 6a,b). Application of 10 μM Val also did not affect the cytoplasmic pH (Supplementary Fig. 6d,e), but failed to produce reliable DiBAC4(3) staining in *Arabidopsis* root epidermal cells, despite a clear phenotype apparent from the transmitted light images (Supplementary Fig. 6c). Val (10 μM, 30 min) and 100 mM KCl application failed to inhibit FM4-64 uptake (Fig. 4c,d; Supplementary Fig. 6c), yet Val abolished staining of mitochondria with MitoTracker Red CM-H2XRos, indicative of its action as ionophore across cellular membranes (Supplementary Fig. 6f). Correspondingly, all three protonophores CCCP, TyrA23, and ES9 inhibited FM4-64 uptake in the presence of 100 mM KCl (Fig. 4c,d) at pH 5.5. Increasing pH gradually in the presence of 100 mM KCl delayed, but did not prevent, FM4-64 uptake in the presence of TyrA23 and CCCP (Supplementary Fig. 6g). Both CCCP and TyrA23 inhibited FM4-64 uptake at medium pH <6.5 with or without the presence of 100 mM KCl (Fig. 4c; Supplementary Fig. 6g).

Altogether these data suggest that the very fast CME block by the protonophores is not due to energy depletion, or dissipation of membrane potential, but largely to cytoplasmic acidification.

## Discussion

Here, we have identified and characterized the primary mode of action of a small molecule ES9, which inhibits CME in *Arabidopsis*, HeLa cells and *Drosophila* neurons. ES9 affected energy metabolism in all systems in the same manner as the known ATP production inhibitors. Surprisingly TyrA23, a known CME inhibitor (Supplementary Data 1), also reduced the cellular ATP levels. Both ES9 and TyrA23 displayed similar effects as CCCP on mitochondrial respiration and uncoupled oxidation and phosphorylation in isolated *Arabidopsis* mitochondria. Likewise, treatments with ES9 or TyrA23 affected the staining of several mitochondrial dyes *in vivo*, indicating that they might depolarize the mitochondrial membranes in different systems. The similarities in phenotypes exhibited by ES9, TyrA23, and CCCP suggested that these compounds have similar mode of action that differs from other mitochondrial inhibitors, such as AA or oligomycin. Indeed, whereas AA and oligomycin are specific inhibitors of ATP production^32^,^33^, CCCP is a known protonophore and non-specific ATP production inhibitor, because it also depolarizes other membranes in the cell^41^. As ES9 and TyrA23 mimicked the CCCP action in almost all aspects tested, it might reasonably be presumed that ES9 and TyrA23 also act as protonophores, an assumption supported by the chemical characteristics of each compound. Like CCCP, both ES9 and TyrA23 are weak acids (sulfonamide and phenol, respectively) that can dissociate into a proton and a stabilized, lipophilic anionic form at physiological pH, thus fitting the classical biochemical description of uncouplers (Supplementary Fig. 7). Although TyrA23 is quite hydrophilic relative to other uncouplers, its anionic form is probably stabilized by an intramolecular hydrogen bond, a known chemical feature in uncouplers that further increases anion lipophilicity, and thus, membrane permeability^47^. In contrast, the TyrA51 analogue, which does not inhibit CME in plant cells, is more hydrophilic, which may explain its apparent less pronounced effects on mitochondrial function and cytoplasmic acidification.

Because ATP is required for CME in mammalian cells^31^, ES9 and TyrA23 might efficiently block CME through ATP depletion. However, although oligomycin and AA both reduced cellular ATP in *Arabidopsis* suspension cell cultures, they did not block endocytosis in root cells to the same extent as ES9 or TyrA23. Previously, a <5% reduction in ATP content with AA has been shown to be sufficient to prevent endocytosis of the epidermal growth factor and the translocation of β-adrenergic receptors^30^. It is possible that the length of treatments with the metabolic inhibitors in root cells used in our study did not reduce efficiently the ATP levels to concentrations low enough to block endocytosis. However, in yeast cells, depletion of ATP to 1–3% of the normal level did not inhibit CME, but affected vacuolar transport^48^, suggesting that different eukaryotic systems may have different ATP requirements for CME. Moreover, the inhibitory effects of ES9 and TyrA23 on CME were rapid (<1–3 min) and faster than the observed drop in ATP level, implying that the endocytosis block induced by these compounds cannot be attributed to ATP depletion alone. This conclusion is consistent with the observations in *Drosophila*, in which defects in mitochondrial function and reduction in ATP levels have no clear implications in synaptic vesicle recycling except under intense stimulation of neurons to mobilize reserve pool vesicles^40^. In agreement, the mitochondrial uncoupler CCCP, like oligomycin and AA, reduced the FM1-43 dye uptake but it did not result in the accumulation of huge membrane inclusions as seen when the CHC^19^ or dynamin^20^ functions are impaired.

Our data show that cytoplasmic acidification (pH<6.5), but not dissipated plasma membrane potential, is the primary cause for CME inhibition in plant cells triggered by the protonophores. In mammalian systems, acidification of the cytoplasm with weak acids strongly reduces CME of transferrin and the epidermal growth factor but has little effect on the uptake of some toxins and fluid phase endocytosis^45^,^49^. Mitochondrial uncouplers of oxidative phosphorylation are also weak acids that, due to their ability to cross membranes in both their protonated and unprotonated form, should be much more effective in perturbing cytoplasmic pH than weak acids that cross a membrane mainly in their protonated form. Indeed, uncouplers like CCCP have activity at other membranes, leading to cell acidification and cytotoxicity^41^,^50^,^51^. Protonophore uncouplers are commonly used to alter the pH of the endocytic vesicles and lysosomes in mammalian cells^41^,^52^. In contrast, the use of protonophore uncouplers to study CME is less frequent because the effects of these compounds across different experimental systems are variable and, thus, more difficult to interpret. Whereas CCCP blocks clathrin-dependent transferrin-mediated iron uptake in reticulocytes^49^, CCCP has no impact on the internalization of fluorescein isothiocyanate-dextran in yeast cells^48^. A possible explanation for the differential effects of protonophore uncouplers on CME might be the difference in cellular environments of the various experimental systems, as supported by our observations that the pH of the incubation media influenced the effect of the uncouplers on the CME. Accordingly, the cytoplasmic pH measurements in *Arabidopsis* suggest that the main proton flow into the cytoplasm is established over the plasma membrane, although we cannot rule out a contribution from the vacuole and other organelles. Hence, protonophores are expected to induce more drastic effects in *Arabidopsis* than in mammalian systems, because the pH of the growth medium differs by more than one pH unit. Interestingly, an increase in pH of the extracellular environment has been shown to reduce the protonophore action of carbonyl cyanide-*p*-trifluoromethoxyphenylhydrazone^50^, a small molecule uncoupler similar to CCCP, at the plasma membrane^41^, corresponding to the observed limitation of CCCP to inhibit FM4-64 uptake at neutral pH in plant cells (Fig. 4c,d). Further evidence in support of the cell-wide effects of CCCP, ES9, and TyrA23, is the inability of the mitochondrial electron transport chain inhibitor AA^32^ to inhibit FM4-64 uptake, to block Golgi and TGN/EE dynamics, as well as to cause cytoplasmic acidification. Acidification of the cytoplasm in mammalian cells did not reduce the number of clathrin-coated pits at the cell surface but interfered with the budding of clathrin-coated vesicles from the plasma membrane as well as from the TGN^45^,^53^. Interestingly, it has been proposed that cytoplasmic acidification is required to induce the curvature of the clathrin lattice^54^. Whether acidification of the plant cell cytoplasm has a similar effect on the clathrin lattice structure remains to be determined. Ultrastructural analysis of high-pressure frozen and freeze-substituted *Arabidopsis* root cells treated with CCCP and ES9 did not reveal any detectable morphological changes in either the plasma membrane, the mitochondria, or the number of clathrin-coated pits, although clathrin-coated pits are scarcely detected in these tissues^55^ (ES9 treatment: 1 clathrin-coated pit/87 μm plasma membrane; DMSO control: 1 clathrin-coated pit/117 μm of plasma membrane) (Supplementary Fig. 8a,b). Experiments in living plant cells indicated that cytosolic acidification dramatically impaired the dynamics of clathrin (CHC, CLC) and associated adaptor proteins (AP-2, TPC) but did not affect the dynamics of the dynamin-related protein, DRPA1. These observations support the hypothesis that, as in mammalian cells^45^,^53^, cytoplasmic acidification may possibly impair the scission of clathrin-coated pits in plants. In addition to inhibiting CME dynamics the protonophores, but not the membrane dissipation agents, caused a reduction in PI(4,5)P_2_ levels as detected by the PI(4,5)P_2_ biosensor and direct measurement of the phosphoinositides (Supplementary Fig. 2). The loss of PI(4,5)P_2_ upon protonophores application correlated with a strong *in vitro* pH dependency of the two PI(4,5)P_2_-forming enzymes from *Arabidopsis*, PIP5K1 and PIP5K2 kinases (Supplementary Fig. 2d), which have been demonstrated to control CME in *Arabidopsis* root cells^56^. In this context we cannot exclude that ATP depletion also affected the activation of the lipid kinases because treatment with AA in time course experiments also reduced PI(4,5)P_2_ levels (Supplementary Fig. 2c). As PI(4,5)P_2_ is an important lipid-binding partner of endocytic proteins in animals^57^, and is required for CME in plants^56^, we can speculate that a reduction in PI(4,5)P_2_ levels will prevent further recruitment of the CME machinery. Similarly, in mammalian cells, it was shown that the depletion of PI(4,5)P_2_ resulted in a loss of clathrin-coat components from the plasma membrane^58^.

Overall our data support a protonophore-induced inhibition of endocytosis by ES9 and TyrA23. However, the ES9 activities differed from the general protonophore effects in terms of synaptic vesicle recycling in *Drosophila*, where the phenotype triggered by ES9 resembled defects in clathrin or dynamin functions^19^,^20^. The inhibitory effect of ES9 on FM4-64 uptake at more alkaline apoplastic pH was retained, suggesting that, besides acidification, ES9 might also inhibit endocytosis through direct interaction with the CME machinery. This however remains to be determined. Our ultrastructural analysis of compound-treated *Arabidopsis* root cells revealed that both ES9 and CCCP induced morphological alterations of the Golgi apparatus and the TGN/EE (Supplementary Fig. 8c) similar to the reported effects of CCCP in mammalian cells^59^,^60^. Interestingly, only the ES9 treatment induced the formation of Golgi-endoplasmic reticulum (ER) hybrid compartments (Supplementary Fig. 8c), indicative of a non-functional coat protein I trafficking^61^. This observation again points towards targets of ES9 that are independent from its proton gradient uncoupling activity.

To date, TyrA23 is the most commonly used CME inhibitor in plants (Supplementary Data 1). This is probably because other inhibitors of this process in mammalian cells, such as dynasore, do not appear to function very efficiently in plants. Given our results on the protonophoric characteristics of TyrA23, the data obtained with this inhibitor in plant cells should be evaluated in light of an acidification-induced block of endocytosis, rather than interference between cargo and adaptor molecules. The general endocytosis block caused by TyrA23 also argues against the latter. TyrA23 still might affect cargo recruitment by AP-2 in mammalian cells, but in plants the essential biochemical proof is still lacking, as subtle differences in amino acid composition and tertiary or quaternary AP-2 structure might compromise TyrA23 binding. Nonetheless, additional effects of TyrA23 on inhibition of CME through cargo recognition cannot be excluded at this point. However, the protonophore activity of TyrA23 overrides any specificity at the level of cargo recruitment by AP-2, because cytoplasmic acidification impairs CME severely. Unless a TyrA23 analogue is developed that cannot dissipate proton gradients and can still interfere with cargo recruitment, the use of TyrA23 to specifically inhibit cargo recruitment to AP-2 should be avoided, at least in plants.

## Methods

### Plant material and growth conditions

*Arabidopsis thaliana* (L.) Heynh. (accession Columbia-0 [Col-0]) seedlings and other lines were stratified for 2 days at 4 °C and grown vertically on agar plates containing half-strength Murashige and Skoog (½ MS) medium supplemented with 1% (w/v) sucrose for 5 days at 22 °C in a 16-h light/8-h dark cycle, prior to use. *Arabidopsis* seedlings were used as control or for labelling of mitochondria and acidic compartments. The following marker lines were used: pTML-TML-GFP/*tml-1* (ref. 3), pTPLATE-TPLATE-GFP/*tplate* (TPL-GFP)^3^, pCLC1-CLC1-GFP/Col-0, pCLC2-CLC2-GFP/Col-0, pCLC3-CLC3-GFP/Col-0, pRPS5A-CHC1-GFP/Col-0, p35S-AP2A1-GFP/Col-0 (ref. 21), pAP2M-AP2M-GFP/*ap2m*^22^, pAP2S-AP2S-GFP/*ap2s*^23^, mRFP-DRP1A^24^, ST-mRFP^26^, VHA-a1-mRFP^27^, P24Y^25^ and P21Y^25^, GFP–VAMP727 (ref. 62), and ABD2-GFP (ref. 29).

### Expression of GFP-tagged CHC1 and CLC in *Arabidopsis*

Entry clones pDONRRP4-1R_RPS5A^21^, pDONRRP2R-P3_GFPstop, and pDONR211 entry of CHC1 (At3g11130) were used together with pK7m34GW in multisite Gateway reactions (Life Technologies) to yield the pRPS5A-CHC1-GFP construct. For cloning in pDONR221, the CHC1 was amplified with primers CHC1fwd and CHC1rev-nostop. To generate the pCLC2-CLC2-GFP construct, a genomic fragment containing CLC2 (At2g20760) and ∼1.2 kb of DNA upstream of the putative ATG start site of CLC2 was amplified by PCR with the bacterial artificial chromosome (BAC) DNA F5H14 as a template and *NsiI*-forward and *SacI*-reverse primers. The PCR product pCLC2-CLC2 was cloned into pGEM-T EASY (Promega) by site-directed mutagenesis (New England Biolabs). On the resulting construct (pSB1107), the adenosine of the putative ATG start site of At2g20770, which is located within the 1.2 kb CLC2 upstream sequence, was deleted and transcribed on the opposite strand of CLC2. The resulting construct (pSB1109) was digested with *NsiI* and *SacI* and sub-cloned into the *PstI*-*SacI* sites of the plant expression vector pSB384 (ref. 63) to generate pSB1112. The construct containing the C-terminally GFP-tagged CLC1 (At2g40060) (pCLC1-CLC-GFP) had been described previously^64^ To generate the pCLC3-CLC3-GFP construct, a genomic fragment containing CLC3 (At3g51890) and ∼1.5 kb of DNA upstream of the putative CLC3 start site, was PCR amplified with Col0 genomic DNA as a template and *SalI*-forward and *SacI*-reverse primers. The pCLC3-CLC3 PCR product was cloned into pGEM-T Easy (Promega) for DNA sequence analysis. The resulting construct (pSB1399) was sub-cloned as *SalI*-*SacI* fragment into the *PstI*-*SacI* restriction sites of pSB384 to generate pSB1404. The pCLC-CLC-GFP and pRPS5A-CHC1-GFP constructs were transformed into *Arabidopsis* (accession Col-0) wild type plants by the *Agrobacterium tumefaciens*-mediated floral dip method^65^. The pCLC-CLC-GFP and pRPS5A-CHC1-GFP transgenic plants were selected by growth on ½ MS agar (0.6% [w/v]) plates containing 50 mg ml^−1^ kanamycin. Primers used during the cloning procedures are listed in Supplementary Table 1.

### Chemical treatments and imaging in *Arabidopsis*

TyrphostinA23, tyrphostinA51, oligomycin, antimycinA, carbonyl cyanide 3-chlorophenylhydrazone, brefeldin A, valinomycin, Bis(1,3-dibutylbarbituric acid) trimethine oxonol (DiBAC4(3)) and fluorescein diacetate (all from Sigma-Aldrich) were dissolved in DMSO (Sigma-Aldrich), except oligomycin and valinomycin in ethanol. ES9 was acquired through Chembridge (http://www.chembridge.com/). Endocytosis was visualized with application of 2 μM *N*-(3-triethylammoniumpropyl)-4-(6-(4-(diethylamino) phenyl) hexatrienyl) pyridinium dibromide (FM4-64, Life Technologies). For staining of mitochondria, seedlings were first treated for 30 min in 1/4; MS (a 1:1 dilution of ½ MS with water) with the respective small molecules. Subsequently, seedlings were transferred to liquid 1/4; MS with 250 nM MitoTracker Red CM-H2XRos (Molecular Probes, Life Technologies) and the respective small molecules for an additional 30 min. Similarly, acidic compartments were labelled with Lyso Tracker Red DND 99 (Molecular Probes, Life Technologies) by treating seedlings with 250 nM Lyso Tracker Red DND 99 in liquid 1/4 MS for 30 min. The small molecule treatment started by addition of the small molecule at the respective concentration to seedlings in the solution-containing Lyso Tracker Red DND 99 for another 30 min. Seedlings were stained with DiBAC4(3) (10 μM) for 30 min together with the respective treatments. Seedlings were imaged on an FV10 ASW confocal laser scanning microscope (Olympus) with a 60 × water immersion lens (numerical aperture [NA] 1.2) and 3 × digital zoom. Endocytic foci *in vivo* were measured with an Ultra View Vox Spinning disc confocal imaging system (PerkinElmer), running on the Volocity software package mounted on an Eclipse Ti inverted microscope (Nikon) with a Plan Apo Lambda 100 × oil NA 1.45) corrected lens and third-generation perfect focus system (PFSIII) for Z-drift compensation. Time series were acquired at two time points per second intervals for 5 min. Excitation was done with a solid-state 488 nm DPSS laser (50 mW) and images were acquired with an ImagEM C9100-13 512 × 512 back-illuminated (16 × 16 μm pixel size) electron microscopy charge-coupled device camera (Hamamatsu Phototonics). Images were processed with the ImageJ software packages (Fiji). For kymographs, background was subtracted with a rolling ball radius of 50 pixels and a walking average of 2 was applied. Kymographs were generated with a line thickness of 3. Images were converted to 8-bit in ImageJ for FM4-64 signal intensity measurements. Regions of interest (ROIs) were selected based on the plasma membrane or cytosol localization. Histograms listing all intensity values per ROI were generated and the averages of the 100 most intense pixels were used for calculations.

### ATP measurements

Wild type PSB-D *Arabidopsis* cell cultures were used and maintained as described before^66^. Three-day-old cell cultures were diluted 100 times and mixed thoroughly before distribution of 95 μl in 96-well plates. Subsequently, 5 μl of a 1/50 dilution of the small molecule stock solution (× 1,000) in MS medium with Minimal Organics medium was added to cells (final dilution of × 1,000) with a Freedom EVO robot (Tecan). ATP levels were detected by addition of 80 μl of the ATPlite 1step Luminescence Assay System (PerkinElmer) after incubation of cells in the presence of small molecules for the indicated time. Fluorescence was measured with a EnVision 2104 Multilabel Reader (PerkinElmer) with the Wallac EnVision manager software package. ATP lite luminescence was detected with the ultra-sensitive luminescence technology. FDA stock solution (2% [w/v] FDA in acetone) was diluted 100 × in target medium and 5 μl was added to 95 μl cell culture. Fluorescence was detected with an excitation at 485 nm (band width 14 nm) and emission at 535 nm (band width 25 nm). The same procedure applied for the Drosophila Schneider 2 (S2) cell suspension cultures that were maintained in Sf-900 II serum-free medium (Gibco) at 25 °C without CO_2_ and distributed in 96-well plates for analysis (95 μl, 25,000 cells). The Jurkat cells were maintained at 37 °C and 5% CO_2_ in RPMI1640 (Gibco) medium and distributed in a similar manner as the S2 cells.

### Measurement of respiratory control ratios

Mitochondria from *Arabidopsis* leaves were isolated following the previously described method A^67^. Briefly, 5 g of leaves were ground with a mortar and a pestle, filtered through a 20 μm nylon net, and crude mitochondria were concentrated with differential centrifugations. Mitochondria were kept on ice prior to respiratory measurements. Respiration was measured accordingly^67^ with a clark-type oxygen electrode (Hansatech) at 25 °C and recorded with a sekonic SS-250F recorder. Dioxygen concentration in air-saturated water was established at 240 μM and a final volume of only 0.5 ml was used in the cuvette. A mix of malate/glutamate (0.1 M/1 M) was chosen as substrate for the mETC and was injected to a final dilution of 1/100. Mitochondrial respiration was stimulated by injection of ADP (10 mM) to a final dilution of 1/100. TyrA23, TyrA51, and CCCP were solubilized with DMSO and measurements of only 5 μl DMSO injected at the beginning, the middle, and at the end of the treatments were used as controls. Independent extractions were repeated 4–6 times with consistent results. Traces shown are actual traces obtained with the recorder, scanned, and digitalized.

### Phospholipid level determination in *Arabidopsis*

Phospholipid levels were measured as described^68^. Briefly, 7-day-old seedlings grown on 1% (w/v) agar plates containing ½MS medium supplemented with 0.5% (w/v) sucrose, 0.1% (v/v) 2-(*N*-morpholino)ethane sulfonic acid (MES) and pH 5.7 (KOH), were used for the experiment. Three seedlings per sample were metabolically labelled by flotation overnight in continuous light in 200 μl 2.5 mM MES buffer pH 5.7 (KOH) and 1 mM KCl, containing 10 μCi of carrier-free [^32^P] PO_4_^3−^. Seedlings were treated by addition of 200 μl MES buffer with double concentrations of the various inhibitors or DMSO as control. Treatments were stopped at the indicated times by adding 5% (v/v) perchloric acid. The lipids were extracted and analysed by thin-layer chromatography^68^. Radioactivity was visualized and quantified by phosphoimaging (Typhoon FLA 7000; GE Healthcare). Each treatment was done 3–6 times and repeated twice independently. Lipids levels were normalized with the radioactivity signal of the total lipid sample. N-fold change and corresponding fractional standard deviation were calculated by setting the respective control treatment to 1. Statistical differences between treatments were calculated by performing independent samples *t*-tests in IBM SPSS Statistics (version 21).

### PIP5K1 and PIP5K2 protein expression and enrichment

PIP5K1 and PIP5K2 were recombinantly expressed as maltose-binding protein (MBP) fusions from pMALc5g plasmids (New England Biolabs, Ipswich, MA, USA) in *Escherichia coli* Rosetta2 cells. Starter cultures in 50 ml of 2YT media were inoculated with single colonies and grown at 30 °C overnight with shaking at 200 r.p.m. Expression cultures were inoculated at an OD_600_ of 0.1 and grown in 300 ml of 2YT media (1.6% [w/v] peptone, 1% [w/v] yeast extract, 0.5% [w/v] NaCl) in baffled flasks at 37 °C with shaking at 95 r.p.m. MBP-PIP5K1 expression was induced with 1 mM isopropyl-1-thio-β-D-galactopyranoside (IPTG) at an OD_600_ of 1.5; MBP-PIP5K2 expression was induced with IPTG at an OD_600_ of 0.7. After induction the cultures were shaken at 28 °C and 95 r.p.m. for 4 h. Cells were subsequently harvested by centrifugation for 20 min at 10,000*g*. Bacterial pellets were resuspended in 40 ml of 20 mM Tris-HCl, pH 7.5, 200 mM NaCl, 1 mM EDTA (buffer A), containing protease inhibitor cocktail (Sigma-Aldrich) and 1 mg ml^−1^ lysozyme (Serva Electrophoresis). After incubation on ice for 1 h, cells were further disrupted using a french-press (Gaulin, APV Homogeniser GmbH) at 1,200 bar. Membrane particles were removed by centrifugation for 20 min at 20,000*g* and 4 °C and the supernatant was kept cold during enzyme purification. MBP-PIP5K1 and MBP-PIP5K2 were enriched with an äkta-protein purification system (äkta FPLC, GE Healthcare Life Sciences). Lysates were applied with a flow rate of 1 ml min^−1^ onto a 5 ml MBP-Trap column (GE Healthcare Life Sciences) equilibrated with buffer A. The column was washed with buffer A. Bound protein was eluted with 10 mM maltose in buffer A at a flow rate of 1 ml min^−1^.

### *In vitro* test for PI4P 5-kinase activity

MBP-PIP5K1 and MBP-PIP5K2 activities were tested with 1 μg of enriched protein. To assay for pH-dependent lipid kinase activity, a three-component system buffer of 50 mM acetic acid, 50 mM MES and 100 mM Tris was used. The pH of the buffer was adjusted with HCl or NaOH by one-unit increments from pH 3 to 10. After incubation at room temperature for 1 h, lipid products were extracted as previously described^69^. The extracted lipids were dried, redissolved in 20 μl chloroform, and were then separated by thin-layer chromatography using silica S60 plates (Merck) and chloroform:methanol:ammonium hydroxide:water (45:45:4:11; v/v/v/v) as developing solvent. Radiolabelled lipids were visualized by exposing a phosphorimager screen (BAS-MP 2040s, Fuji) and the extent of 33P-incorporation was quantified by phosphorimaging (BAS-1500).

### TEM of *Arabidopsis* root tips

*Arabidopsis* root tips from 5-day-old seedlings were submerged in 200 mM sucrose, 10 mM trehalose, and 10 mM Tris buffer, pH 6.6 (ref. 70), transferred into planchettes (3.0 × 0.5 mm, Al, type A and B; Leica Microsystems), and frozen in a high-pressure freezer HPM100 (Leica Microsystems). Freeze substitution was done in a EM AFS2 unit (Leica Microsystems) in dry acetone supplemented with 0.4% (v/v) uranyl acetate at −85 °C for 16 h, followed by 5 h warm-up to −50 °C. After washing with 100% (v/v) ethanol for 60 min, samples were infiltrated and embedded in Lowicryl HM20 resin at −50 °C (intermediate steps of 30, 50, 70% HM20 in ethanol, 1 h each). The resin was polymerized with ultraviolet light for 3 days in the freeze substitution unit. Ultrathin sections were cut on a ultramicrotome UC7 (Leica Microsystems) and post-stained with aqueous uranyl acetate/lead citrate. Sections were examined by JEM 1230 (JEOL) or CM100 (Philips) transmission electron microscopes, both operating at 80 kV. Micrographs were recorded with a MSC 600CW (Gatan) digital camera or by scanning negatives (MACO EM films TYP S, 6.5 × 9 cm, ES 206) with a Perfection V750 Pro system (Epson).

### pH measurements

For cytosolic pH measurements, 4-day-old UB10:pH-GFP plants^43^ were transferred to 2 ml of liquid ½MS (pH 5.5, 2.15 g l^−1^ MS salts, 0.5 g l^−1^ MES) for 30 min with the indicated drugs or mock (DMSO). Imaging was done with a 700 confocal microscope (Zeiss) with a Plan-Apochromat 20 × /0.8 M27 objective lens. pH-GFP was excited by 405 and 488 nm diode lasers and the emission was collected separately between 500 and 555 nm. The images were evaluated in ImageJ (Fiji) by measuring intensities of both channels in six circulars ROIs per root meristematic zone. Vacuolar pH was measured as described before^44^ for a 2-h incubation with the indicated small molecules.

### Statistical tests and generation of graphs

All statistical tests and graphs other than boxplots were performed and generated using SigmaPlot 13. Boxplots were generated with the online tool BoxPlotR (http://boxplot.tyerslab.com/) from the Tyers and Rappsilber laboratories.

### *Drosophila* genetics

Fly stocks were maintained on standard maize meal and molasses medium and were obtained from the Bloomington Drosophila Stock Center (Indiana University) or from the Leuven Institute for Neurodegenerative Disease (Leuven, Belgium)^20^.

### FM1-43 uptake assay and immunohistochemistry in *Drosophila*

FM1-43 labelling was done as described^71^. Third-instar wild type larvae were dissected in HL-3 and, before a stimulation protocol, flies were incubated for 30 min in HL-3 supplemented with and without 10 μM ES9 or for 10 min in HL-3 supplemented with 10 μM CCCP or mock (1% [v/v] DMSO). Next, neurons were stimulated for 5 min in HL-3 with 90 mM KCl and 1.5 mM CaCl_2_ in the presence of FM1-43 (4 μM) (Life Technologies) with or without small molecules. After labelling, the preparation was washed several times with HL-3 supplemented with or without small molecules. Images were captured with a confocal microscope (A1R, Nikon) with a 60 × (NA 1.0) water dipping lens and a 4 × zoom. The boutonic FM1-43 intensity was quantified with ImageJ and corrected for background labelling in the muscles. Immunohistochemistry was performed as described^20^. Larvae were treated in a similar fashion as for the FM1-43 dye uptake assay without the presence of FM1-43. Not-stimulated animals were baded in HL-3 without small molecules. The treated flies were fixed and stained with primary antibodies. Alexa 555-conjugated secondary antibodies or Alexa 488-conjugated secondary antibodies (Life Technologies) were used at 1:1,000. Larvae expressing HA-tagged CHC (HA-Chc/+) were stained with a mouse anti-HA antibody (Covance) at 1:500 and rabbit-anti-α-adaptin antibody^72^ at 1:200 (gift from M. González-Gaitán) and Alexa 555-conjugated secondary antibodies. Larvae expressing PLCδ1-PH-GFP under the control of the neuronal nSybGAL4 promoter (PLCδ1-PH-GFP, nSybGAL4/nSybGAL4)^73^ to visualize the localization of PI(4,5)P_2_ were not double stained. Super-resolution structural illumination microscopy images were acquired on a microscope (Elyra S.1; Carl Zeiss) with a 63 × (NA 1.4) oil lens and three rotations at room temperature. The acquired images were processed and stored with the ZEN 2011 software (Carl Zeiss).

### Phosphatidylinositol analysis in *Drosophila* NMJs

Third-instar larvae expressing PLCδ1-PH-GFP under the control of the neuronal nSybGAL4 promoter (PLCδ1-PH-GFP, nSybGAL4/nSybGAL4) were used to analyse the levels of PI(4,5)P_2_ in the NMJ^73^. Larvae were treated in a similar fashion as for the FM1-43 dye uptake assay without the presence of FM1-43. Not-stimulated animals were baded in HL-3 without small molecules. Next, the treated animals were imaged. Images were captured with a confocal microscope (A1R, Nikon) with a 60 × (NA 1.0) water dipping lens and a 4 × zoom. The average GFP intensity in synaptic boutons of the NMJ were quantified with ImageJ and corrected for background signals.

### TEM of *Drosophila* synaptic boutons of the NMJs

TEM experiments were performed as described^20^. First, third-instar wild type larvae were dissected in HL-3 and, prior to a stimulation protocol, flies were incubated for 30 min in HL-3 supplemented with and without 10 μM ES9. Next, neurons were stimulated for 5 min in HL-3 with 60 mM KCl and 1.5 mM CaCl_2_ with or without small molecules. After labelling, the preparation was washed several times with HL-3 supplemented with or without small molecules. Next, the samples were fixed in 4% paraformaldehyde and 1% glutaraldehyde in 0.1 M Na-Cacodylate buffer (pH 7.4) for 2 h at room temperature and subsequently kept in the fridge overnight. Next, the fixed fillets were washed four times for 15 min in a glass recipient with 0.1 M Na-Cacodylate, pH 7.4 and subsequently osmicated with 2% osmium (OsO_4_/Na-Cacodylate buffer) on ice for 2 h. Subsequently, samples were washed with chilled 0.1 M Na-Cacodylate buffer for 15 min followed by a 15 min wash with ddH_2_O. After the washing step with ddH_2_O, samples were stained with 2% uranyl-acetate and again washed with ddH_2_O before dehydration. After dehydration with an ethanol series samples were placed in propylene oxide for twice 10 min and embedded in Agar 100 (Laborimpex, Agar scientific). Ultrathin sections (70 nm) were cut on an EM UC7 ultratome (Leica), collected on grids (Laborimpex, Agar Scientific), coated with Butvar, and imaged with a JEM 1400 transmission electron microscope (JEOL). Images were acquired at 80 kV with an 11 mega-pixel bottom-mounted camera (Quemesa, Olympus) and iTEM5.2 Software (Olympus) at × 10k and × 20k zooms.

### TMRE labelling in *Drosophila*

TMRE was used to label the mitochondrial membrane potential as described^38^. Third-instar larvae with mitochondria in motor neurons labelled with mitoGFP (D42mitoGFP/+) were dissected in HL-3 and incubated for 30 min in HL-3 supplemented with and without 10 μM ES9. Subsequently, larvae were treated for 15 min with HL-3 medium supplemented with 50 nM TMRE (Abcam) and the appropriate small molecule at room temperature. For CCCP treatment, larvae were incubated for 15 min in HL-3 supplemented with 50 mM TMRE and 10 μM CCCP or mock (1% [v/v] DMSO). Fillets were washed with HL-3 and the appropriate small molecule and images were captured by confocal microscopy (A1R, Nikon) with a 60 × (NA 1.0) water dipping lens. The TMRE labelling intensity was quantified with ImageJ. First, 32-bit image of the mitoGFP channel was thresholded to select the mitochondria of the NMJ. Subsequently, the TMRE labelling intensity was measured in that region.

### HeLa cell cultures

HeLa cells were grown in DMEM (Gibco, Life technologies), supplemented with 10% fetal calf serum (Gibco, Life Technologies) and 1% antibiotics (penicillin/streptomycin) (Gibco, Life Technologies), and maintained in a humidified incubator at 37 °C under a 5% CO_2_ atmosphere.

### Live imaging of HeLa cells

For the microscopy observation of living cells, HeLa cells were placed on a MatTek chamber and incubated overnight in a humidified incubator at 37 °C and under a 5% CO_2_ atmosphere to enable their attachment to the coverslip. All tested compounds were dissolved in DMSO and were diluted to the desired concentration in DMEM medium without fetal calf serum and without phenol red (Gibco, Life Technologies). To study the effect of different compounds on endocytosis in HeLa cells, transferrin from human serum, Alexa Fluor 488 Conjugate (Life Technologies) was implemented. The cells were pre-treated with the compounds for 30 min at 37 °C and a 5% CO_2_ atmosphere, followed by the exchange of the medium with a fresh one, containing the same compound concentration and 25 μg ml^−1^ fluorescently labelled transferrin. Imaging was done with a LSM 700 inverted confocal microscope (Zeiss) with a plan-apochromat 40 × /1.2 water objective lens for a time period of up to 20 min after placing the transferrin-containing solution on the cells. The images were quantified in ImageJ by measuring the signal intensity of the mock-treated (between 0.01% [v/v] and 0.7% [v/v] DMSO) cells and the signal intensity of the compound-treated cells.

### Data availability

The authors declare that all data supporting the findings of this study are available within the article and its Supplementary Information files.

## Additional information

**How to cite this article:** Dejonghe, W. *et al*. Mitochondrial uncouplers inhibit clathrin-mediated endocytosis largely through cytoplasmic acidification. *Nat. Commun.* 7:11710 doi: 10.1038/ncomms11710 (2016).

## Supplementary Material

Supplementary Information Supplementary Figures 1 - 8 and Supplementary Table

Supplementary Data 1 Published work using TyrA23
