# Supplementary material for: Mitochondrial uncouplers inhibit clathrin-mediated endocytosis largely through cytoplasmic acidification
Source: Nat Commun. 2016 Jun 8;7:11710. doi: 10.1038/ncomms11710 (PMC4899852; doi:10.1038/ncomms11710)
Supplement: Supplementary Information — Supplementary Figures 1 - 8 and Supplementary Table [file ncomms11710-s1.pdf]

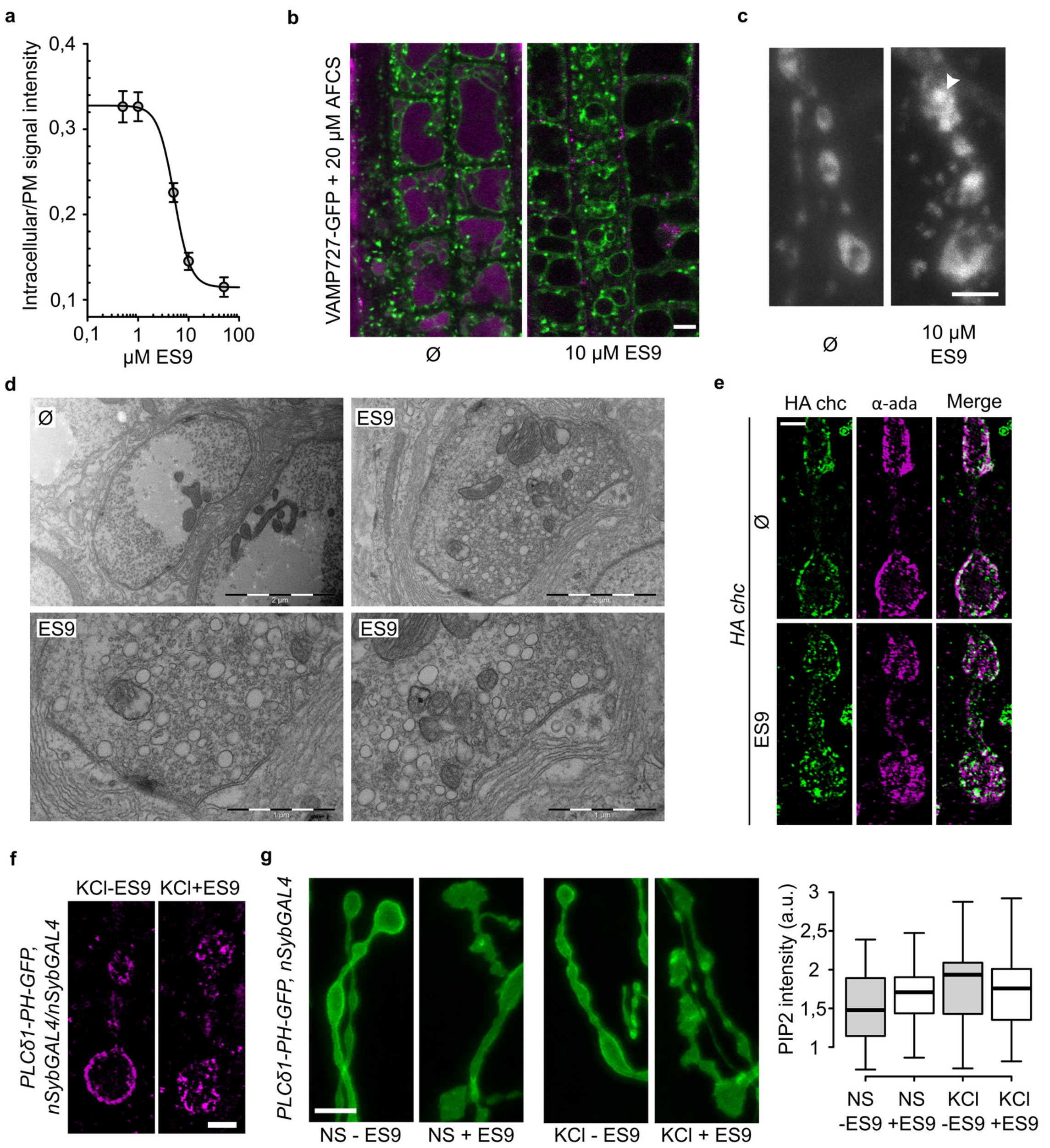

Supplementary Figure 1 | ES9 inhibits clathrin-depended processes in *Arabidopsis* and *Drosophila*. (**a**) Dose-response of ES9 mediated FM4-64 uptake inhibition. Data plotted as the ratio of cytosolic over plasma membrane FM4-64 fluorescence. EC_{50} is 5,16 µM. (**b**) Fluorescently-labelled Alexa fluor 674 castasterone (AFCS) (magenta, 20 µM, 30 min pulse, 20 min chase) uptake assay comparing mock (Ø, DMSO) and 10 µM ES9 (30 min) treatment in VAMP727-GFP-positive (green) *Arabidopsis* root cells. (**c**) FM1-43 labelling of *Drosophila* third instar neuromuscular junctions (NMJs) after 5 min of nerve stimulation with 90 mM KCl, in the control condition (Ø) and after treatment with 10 µM ES9 for 30 min. Note, the presence of large FM1-43-labelled structures (arrowhead). (**d**) Transmission electron microscopy images of *Drosophila* NMJs after 5 min of nerve stimulation with 60 mM KCl, in the control condition (Ø) and after treatment with 10 µM ES9 for 30 min. Note, the increased size of synaptic vesicles in animals treated with ES9. (**e**) Super resolution immunofluorescence images of 90 mM KCl-stimulated *Drosophila* NMJs endogenously expressing HA-tagged clathrin heavy chain (HA-CHC) and stained for alpha-adaptin (AP-2)

and (**f**) PI(4,5)P_2 (visualized with PLCδ1-PH-GFP), comparing mock with 10 µM ES9 (30 min) (**g**) Maximum projections of *Drosophila* NMJs where PI(4,5)P_2 localization was visualized by PLCδ1-PH-GFP in not-stimulated and stimulated animals for 5 min with 90 mM KCl, in the control condition (Ø) and after treatment with 10 µM ES9 for 30 min, together with the quantification of signal intensity. Boxplot centre lines show the medians; box limits indicate the 25th and 75th percentiles as determined by R software; whiskers extend 1.5 times the interquartile range from the 25th and 75th percentiles; outliers are represented by dots. Error bars, s.e.m. n= at least 6 plants per data point in (**a**) and 4 animals in (**g**). Scale bars, 5 µm in (**b**) (**c**) and (**g**), 2 µm in top images of (**d**), (**e**), (**f**), and 1 µm in bottom images of (**d**).

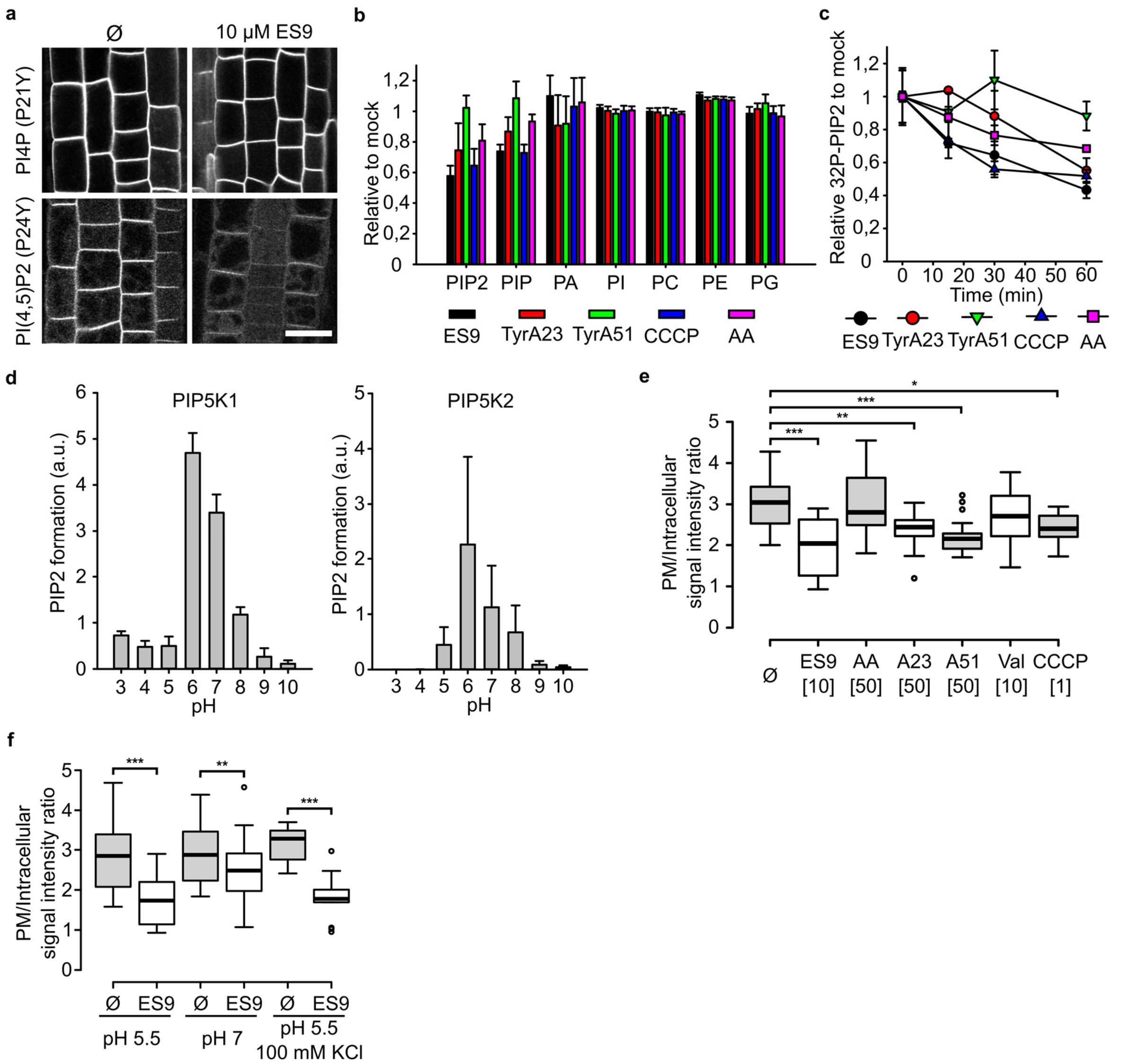

Supplementary Figure 2 | Plasma membrane bound phosphatidylinositols are affected by protonophores. (**a**) Confocal images of *Arabidopsis* roots expressing sensors for phosphatidylinositol 4-phosphate (PI4P, P21Y) and phosphatidylinositol 4,5-bisphosphate (PI(4,5)P_2, P24Y) in mock controls (DMSO, Ø) or upon treatment with 10 µM ES9 for 30 min, revealing a strong reduction in the PI(4,5)P_2 levels. (**b**) Biochemical quantification of ^{32}P-labelled phospholipid levels *in vivo* after 30 min, and (**c**) ^{32}P-PI(4,5P)P_2 levels over time in presence of 10 µM ES9, 50 µM tyrphostinA23 (TyrA23), 50 µM tyrphostinA51 (TyrA51), 2 µM carbonyl cyanide m-chlorophenyl hydrazine (CCCP) and 50 µM antimycin A (AA). Values in (**b**) and (**c**) are relative to mock treatment. Error bars, s.d., n=6 in **(b)** and n=3 in **(c)**. (**d**) *In vitro* analysis of PI(4,5)P_2 formation at different pH by purified recombinant PI4P kinases PIP5K1 and PIP5K2 from *Arabidopsis*. Error bars, s.d., n=3. (**e**) Quantification of plasma membrane association of the P24Y PI(4,5)P_2 marker in presence of mock (Ø, DMSO, n=19 seedlings) ES9 (n=22), TyrA23 (n=21), TyrA51 (n=21), CCCP (n=22), AA (n=19), and valinomycin (Val). (n=23). Concentration is indicated between brackets. (**f**) Quantification of plasma membrane association of the P24Y PI(4,5)P_2 marker for mock (Ø, DMSO) compared to 10 µM ES9 treatments in different media. n, number of seedlings from left to right, 38, 35, 32, 34, 13, and 14. Values in (**e**) and (**f**) are represented as the ratio of plasma membrane over cytosolic fluorescence. Error bars, s.e.m. $^{**}P<0.01$ $^{***}P<0.001$ (Student's *t*-test). A Mann-Whitney Rank Sum Test was performed in case the normality test (Shapiro-Wilk) failed.

Boxplot centre lines show the medians; box limits indicate the 25th and 75th percentiles as determined by R software; whiskers extend 1.5 times the interquartile range from the 25th and 75th percentiles; outliers are represented by dots. Scale bar, 20 μm.

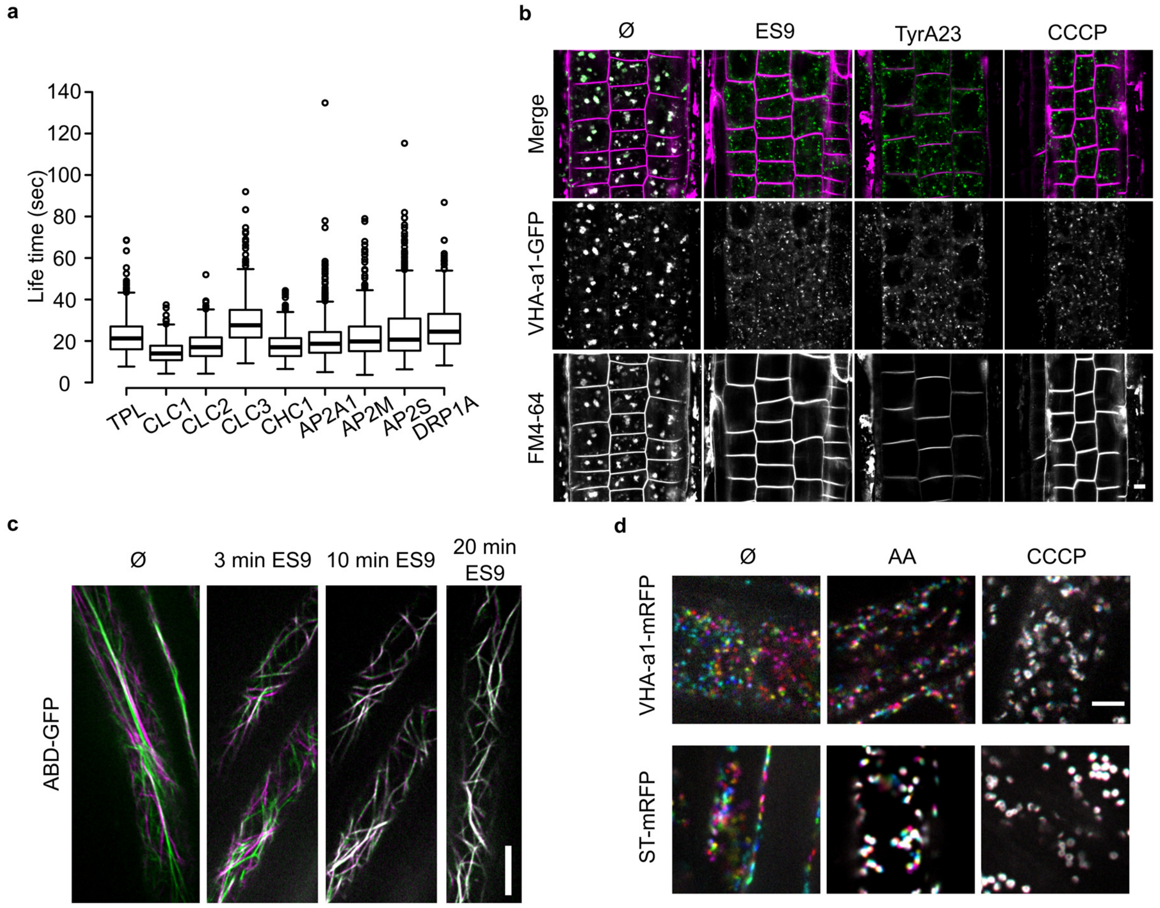

Supplementary Figure 3 | ES9 inhibits Golgi/*trans*-Golgi Network (TGN)/Early Endosome (EE) and actin motility. (**a**) Boxplot representation of kymograph-based life time of foci at the plasma membrane in presence of mock (DMSO) for green fluorescent protein (GFP)-tagged TPLATE, clathrin light chain (CLC), clathrin heavy chain (CHC), the adaptor complex 2 (AP2), represented by the AP2A1, AP2M and AP2S subunits, and the dynamin DRP1A. n=723, 249, 245, 556, 383, 769, 518, 711, and 240 measurements. (**b**) Brefeldin A (BFA, 50 μM, 30 min) body formation is prevented in the presence of 1 μM CCCP, 10 μM ES9 and 50 μM tyrphostinA23 (TyrA23). The VHA-a1-GFP is used to mark the BFA bodies that are agglomerates of the Golgi-TGN/EE network units. Lack of FM4-64 (2 μM) staining of endosomal compartments indicates endocytosis inhibition. (**c**) Actin binding domain (ABD) tagged with GFP indicated lack of actin movement upon ES9 application. Images represent two frames with a 2 min interval for mock (Ø, DMSO) and 10 μM ES9 at different time points. White indicates overlap and represents lack of actin movement. (**d**) Golgi (ST-mRFP) and TGN/EE (VHA-a1-mRFP) visualization in control treatment (Ø, DMSO) and in the presence of 20 μM antimycin (AA) and 1 μM carbonyl cyanide m-chlorophenyl hydrazine (CCCP) (5 min). Images are composed of six differentially coloured images taken with a 10-sec interval. Movement is illustrated by the presence of the different colours, whereas white indicates stationary. Scale bars, 5 μm in (**b**) and (**d**), 10 μm in (**c**).

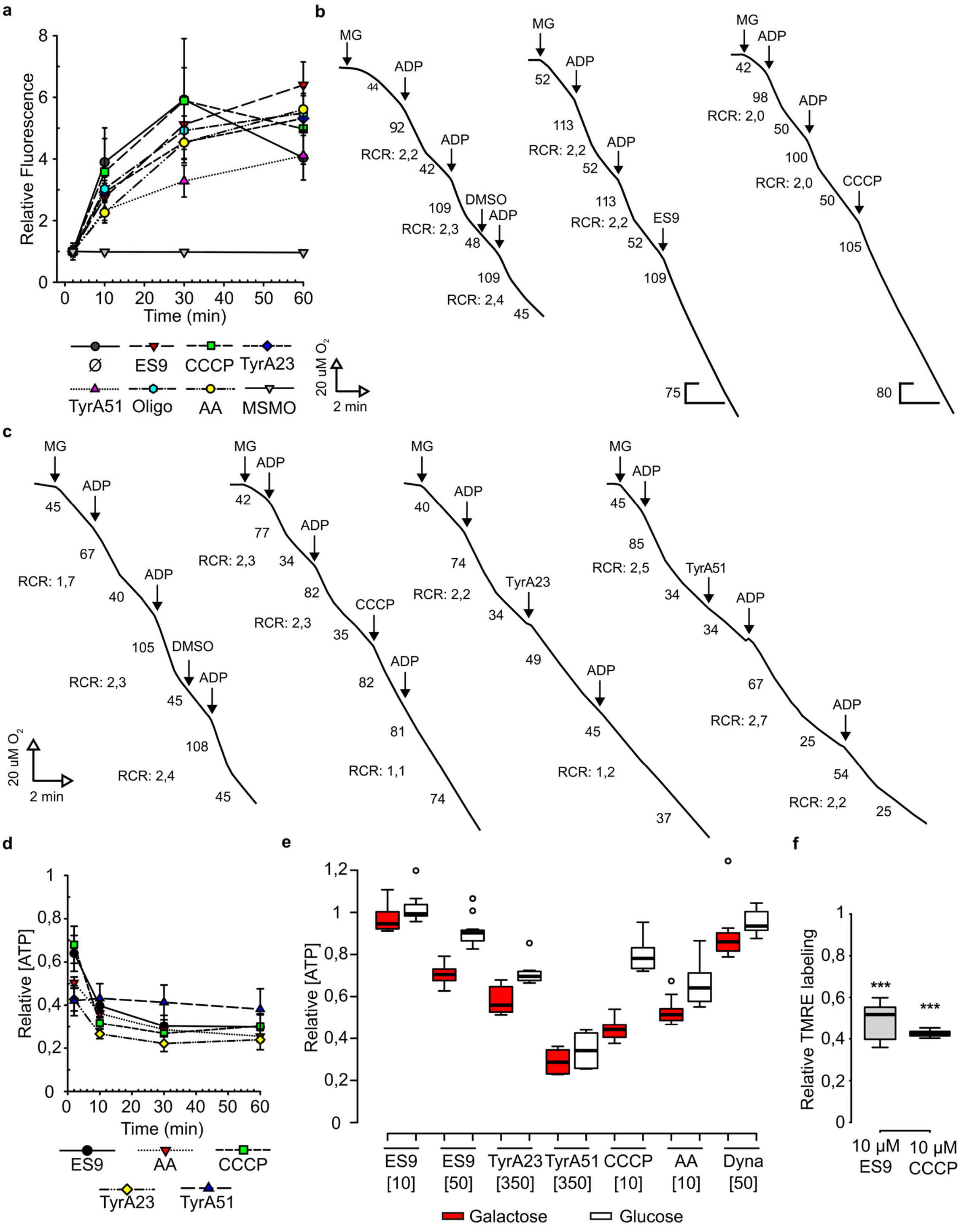

Supplementary Figure 4 | ES9 and TyrA23 affect mitochondrial functions, similar to CCCP. (**a**) Fluorescin diacetate (FDA)-based fluorescent measurements done using PSB-D wild type cell cultures in the presence of 1 µM carbonyl cyanide m-chlorophenyl hydrazine (CCCP), 50 µM tyrphostinA23 (TyrA23), 50 µM tyrphostinA51 (TyrA51), 10 µM ES9, 10 µM oligomycin and 50 µM antimycin A (AA). MSMO, Murashige and Skoog with Minimal Organics medium. Error bars, s.e.m., n=12 measurements. (**b-c**) Measurements of mitochondrial electron transport chain (mETC) respiratory characteristics. Respiration was

stimulated with malate/glutamate (MG) and respiratory rates (values on the left side of traces) are expressed in nmol O_2 min^{-1} mg^{-1} protein. Respiratory control ratios (RCRs) were not affected when treated with mock (DMSO) and 5 µM TyrA51. Clear uncoupling occurred between oxidations and phosphorylations as shown by an RCR drop in the presence of 1 µM ES9, 1 µM CCCP or 5 µM TyrA23. (**d**) Measurement of ATP levels in *Drosophila* S2 cell cultures in the presence of 10 µM CCCP, 10 µM ES9, 50 µM AA, 50 µM TyrA23 and 50 µM TyrA51. ATP levels are depicted relative to mock (DMSO). Error bars, s.d., n=8 measurements. (**e**) Relative ATP concentrations as measured in Jurkat suspension cell cultures after 3 h incubation with 10 and 50 µM ES9, 10 µM CCCP, 350 µM TyrA23 and TyrA51, 10 µM AA and 50 µM dynasore. n= at least 8 measurements. The medium contained either glucose (white plots) or galactose (red plots). Box plot center lines show medians; box limits indicate the 25th and 75th percentiles as determined by R software; whiskers extend 1.5 times the interquartile range from the 25th and 75th percentiles; outliers are represented by dots. (**f**) Quantification of the relative intensity of mitochondrial tetramethylrhodamine ethyl ester (TMRE) labelling in D42mitoGFP-positive mitochondria at the neuromuscular junction of third instar *Drosophila* larvae for ES9 (10 µM, 30 min pretreatment, n= 4 animals) and CCCP treatments (10 µM, 15 min treatment, n=5 animals). ****P*<0.001 (Mann-Whitney Rank Sum Test).

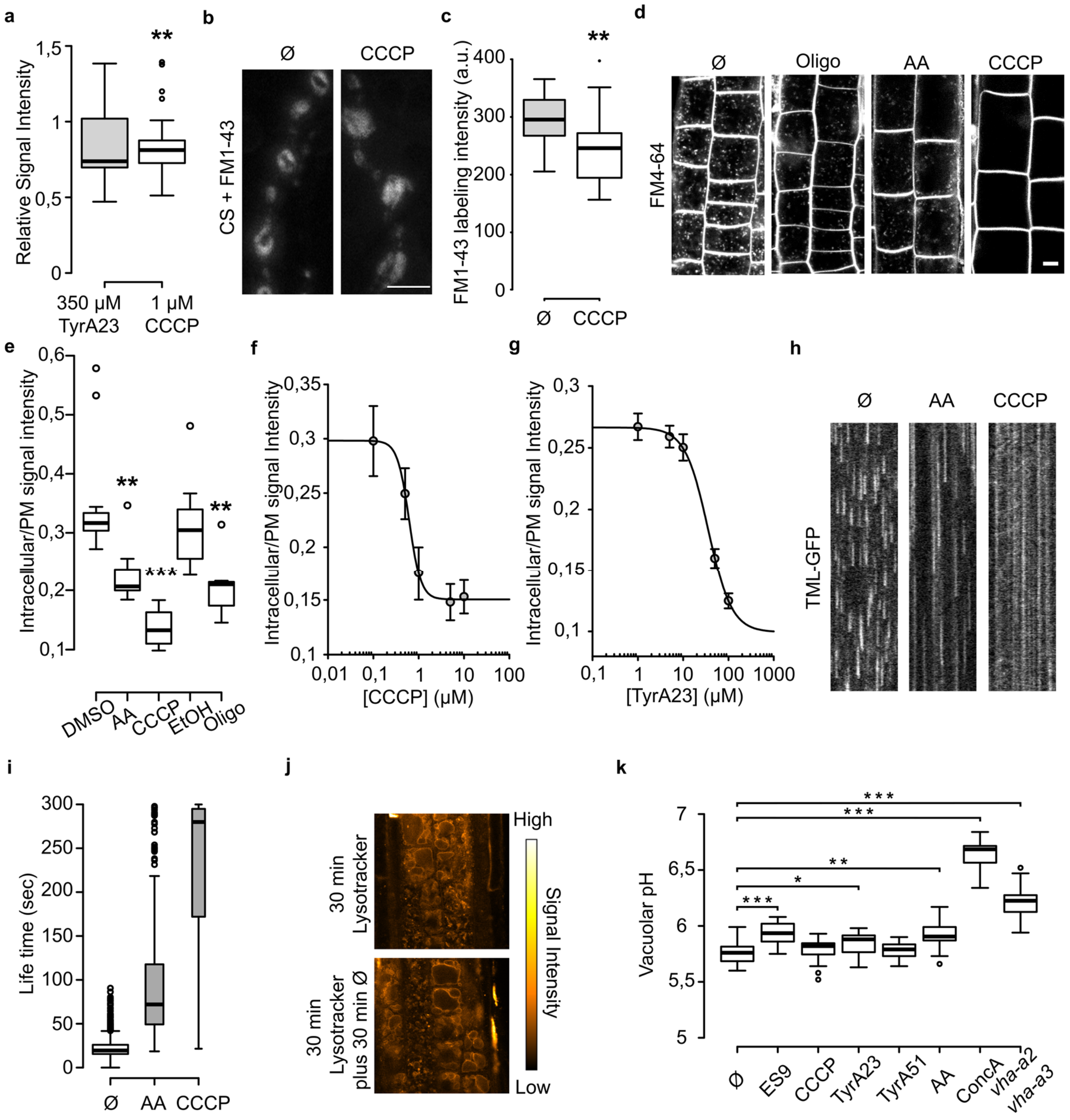

Supplementary Figure 5 | Protonophores inhibit CME. (**a**) Boxplot representation of transferrin uptake in Hela cells in the presence of 1 µM carbonyl cyanide m-chlorophenyl hydrazine (CCCP) and 350 µM tyrphostin A23 (TyrA23) relative to mock (DMSO), n=28 and 25 cells respectively. (**b**) FM1-43 labelling of neuromuscular junction boutons in third instar larvae of *Drosophila* stimulated for 5 min with 90 mM KCl in the presence of the dye, in the mock situation (30 min) and after treatment with 10 µM CCCP for 30 min. (**c**) Signal quantification of (**b**), n=5 and 6 animals respectively. (**d**) FM4-64 uptake (2 µM, 30 min) in *Arabidopsis* root cells in presence of mock (DMSO, EtOH), 50 µM antimycin A (AA), 1 µM CCCP or 50 µM oligomycin (Oligo). Only in the case of oligomycin, cells were pretreated with the compound for 30 min before FM4-64 application. (**e**) Quantification of the plasma membrane-derived FM4-64 signal over the cytoplasmic signal in (**d**), n=13, 9, 10, 10 and 8 seedlings respectively. (**f**) and (**g**) represent dose response curves for CCCP and TyrA23 respectively. IC_{50} for CCCP is 0,61 µM and 36 µM for TyrA23. Data is represented as the ratio of cytoplasmic over plasma membrane signal intensity, n= at least 6 seedlings per data point. (**h**) Kymographs generated from the respective spinning disc movies (2f/sec)

representing a line trace (horizontal axis) over a time period (vertical axis, 5 min), visualizing the life times of endocytic spots labelled by TPLATE muniscin-like (TML)-GFP for the mock treatment, 20 µM AA and 1 µM CCCP. (**i**) Boxplot representation of the measured life times of endocytic spots (n=1646, 423 and 218 measurements), showing a strongly prolonged residence time of the endocytic foci at the plasma membrane in the presence of AA and CCCP. (**j**) Lyso Tracker Red DND 99 labelling is not affected upon mock treatment in *Arabidopsis* seedlings. (**k**) Vacuolar pH measurement for mock treatment (DMSO, Ø), 10 µM ES9, 50 µM TyrA23, 1 µM CCCP, 50 µM TyrA51, 50 µM AA and 1 µM concanamycinA (ConcA) and for mock treatment in the *vha-a2 vha-a3* double mutant background. n=48 for mock treatment, 16 for ConcA treatment, 24 for the mutant, and 32 for all other treatments. $**P < 0.01$ and $***P<0.001$ (Kruskal-Wallis One Way ANOVA on Ranks and pairwise comparison according to Dunn's method). The boxplot centre lines show the medians; box limits indicate the 25th and 75th percentiles as determined by R software; whiskers extend 1.5 times the interquartile range from the 25th and 75th percentiles; outliers are represented by dots. For (**a**), (**c**) and (**e**) $**P<0.01$ (Student's *t*-test or Mann-Whitney Rank Sum Test). Scale bars, 5 µm. Error bars, s.e.m.

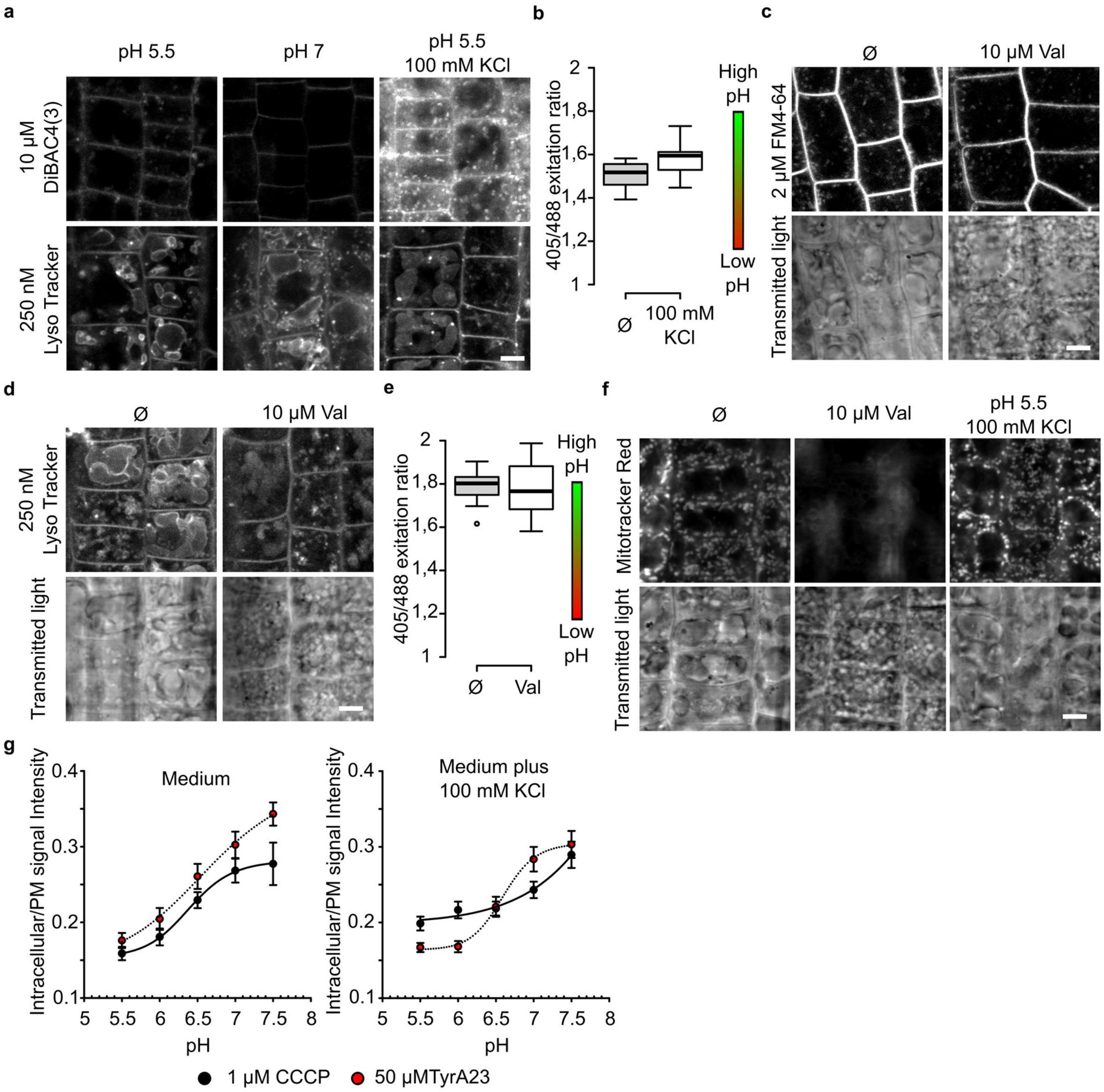

Supplementary Figure 6 | Cytoplasmic acidification is the primary cause of protonophore-mediated CME inhibition. (**a**) DiBAC4(3) staining to indicate change in membrane potential and Lyso Tracker Red DND 99 staining to indicate change in proton distribution in medium at pH 5.5, pH 7 and pH 5.5 supplemented with 100 mM KCl. (**b**) Boxplot representation of cytoplasmic pH measurements using pH-GFP in normal medium (pH 5.5) (n=17 seedlings) and medium supplemented with 100 mM KCl (n=19 seedlings). (**c**) FM4-64 uptake in presence of valinomycin (Val) compared to mock (EtOH, Ø). Note the change in root morphology in the transmitted light images, indicating Val activity. (**d**) Lyso Tracker Red DND 99 staining in presence of Val compared to mock (EtOH, Ø), indicating Val is hardly affecting proton flows in *Arabidopsis* root cells. (**e**) Boxplot representation of cytoplasmic pH measurements using pH-GFP in presence of 10 µM Val compared to mock (EtOH, Ø) n=18 seedlings. (**f**) Mitochondrial staining (125 nM MitoTracker Red CM-H2XRos) of *Arabidopsis* root cells for mock (EtOH, Ø), Val, and mock in medium supplemented with 100 mM KCl. Note the absence of staining in Val-treated roots, indicating ionophoric activity. (**g**) Quantification of FM4-64 uptake in presence of carbonyl cyanide m-chlorophenyl hydrazine (CCCP) and tyrphostinA23 (TyrA23) in normal medium at different

pH and medium supplemented with 100 mM KCl at different pH. Note that in both normal medium and medium supplemented with KCl, CCCP and TyrA23 fail to inhibit FM4-64 uptake at higher pH. Data is represented as the ratio of cytoplasmic over plasma membrane signal intensity in, n= at least 11 seedlings for CCCP and 6 for TyrA23 per data point. The boxplot centre lines show the medians; box limits indicate the 25th and 75th percentiles as determined by R software; whiskers extend 1.5 times the interquartile range from the 25th and 75th percentiles; outliers are represented by dots. Error bars, s.e.m. Scale bars, 5 µm.

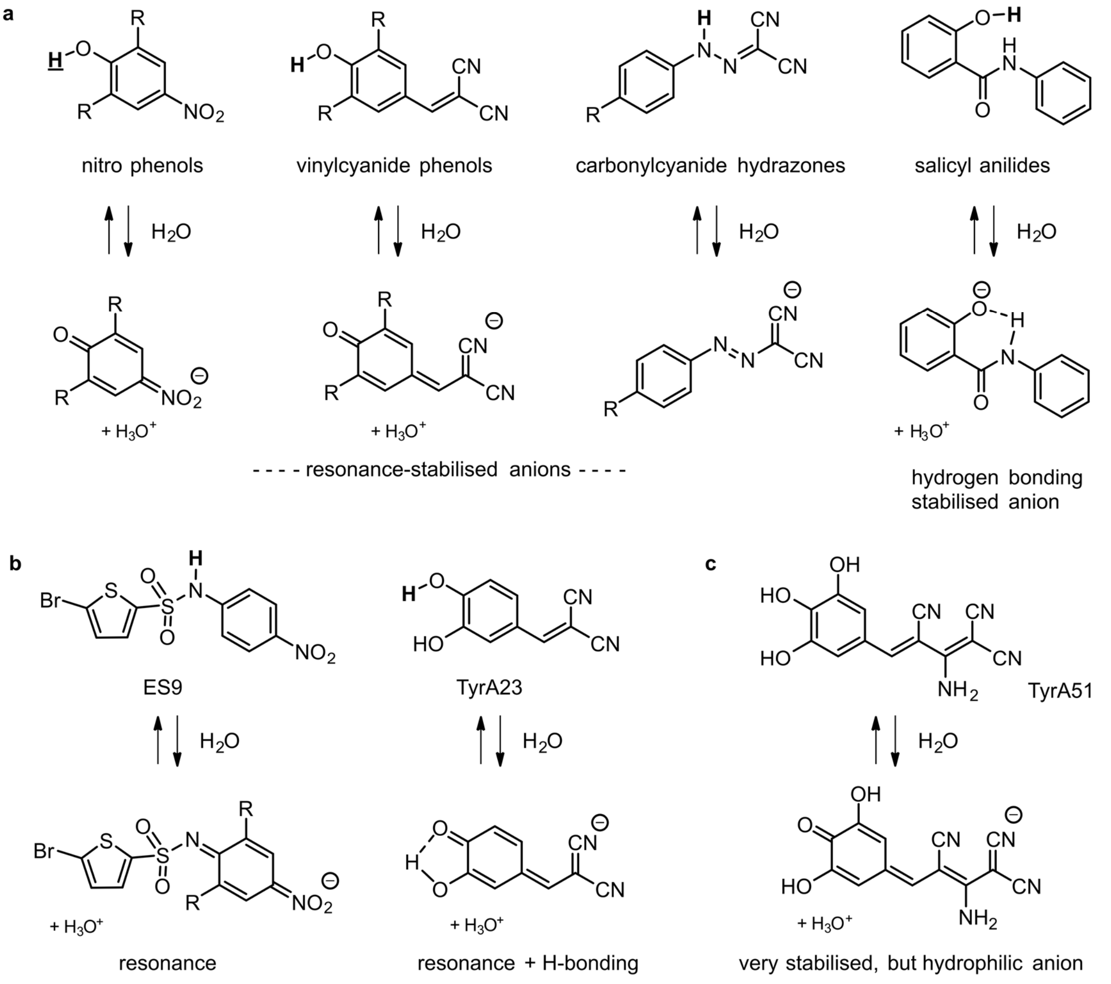

Supplementary Figure 7 | Chemical features of protonophores. (**a**). Four known potent chemical classes of protonophore uncouplers of oxidative phosphorylation releasing lipophilic anions either through electronic conjugation (charge delocalized resonance stabilization) or through intramolecular hydrogen bond stabilization. (**b**). Structures of chemicals found to be protonophore uncouplers in this study, ES9 and tyrphostinA23 (TyrA23) and the structural features of their anions. (**c**). Structure of tyrphostinA51 (TyrA51) which evidently gives a highly stabilized but much less lipophilic anion than the related TyrA23 compound, in agreement with the observed effects upon treatment with both tyrphostins.

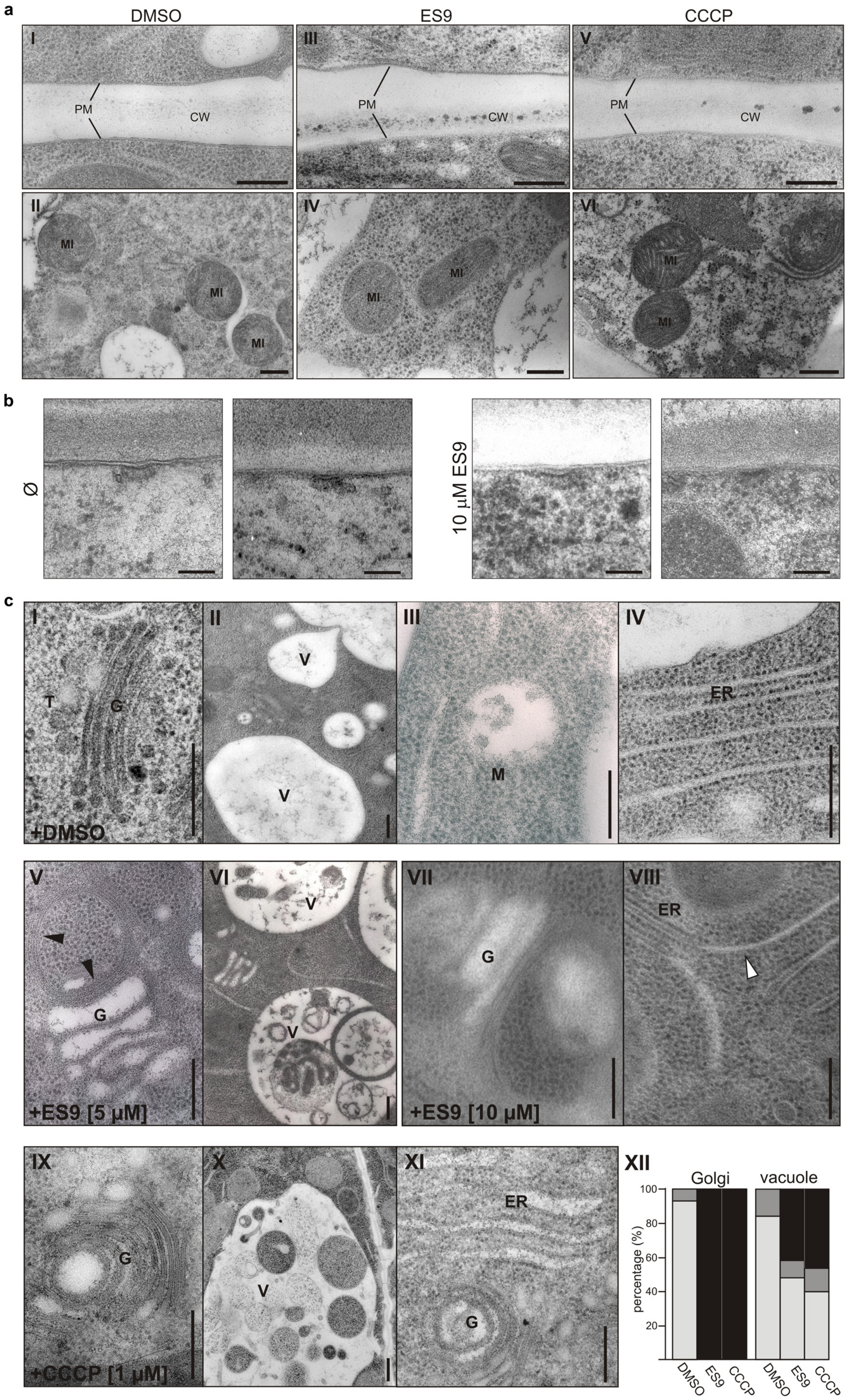
a
DMSO
ES9
CCCP
I
II
III
IV
V
VI
PM
CW
MI
b
Ø
10 μM ES9
c
T
G
V
M
ER
+DMSO
+ES9 [5 μM]
+ES9 [10 μM]
+CCCP [1 μM]
VII
VIII
IX
X
XI
XII
Golgi
vacuole
percentage (%)
100
80
60
40
20
DMSO
ES9
CCCP

Supplementary Figure 8 | Protonophores cause morphological alterations in the endomembrane system. (**a**) Representative transmission electron microscopy (TEM) images of meristematic root cells from 5-day-old *Arabidopsis* seedlings showing that the plasma membrane (PM) (I, III and V) and mitochondria (MI) (II, IV and VI) in mock control (DMSO), ES9 (10 µM, 30 min), or CCCP (1 µM, 30 min) treated samples are morphologically normal. CW, cell wall. (**b**) Visualization of clathrin-coated pits in the PM in mock control (DMSO, Ø) or in ES9 treated samples. (**c**) (I-IV) The Golgi apparatus (G) and the *trans*-Golgi network (T), vacuoles (V), multivesicular bodies (M) and the endoplasmic reticulum (ER) displayed a normal morphology upon 30 min exposure to DMSO. (V, VI), Upon 30 min exposure to 5 µM ES9, the Golgi showed swollen stacks, the TGN (arrowheads) had an aberrant morphology, multivesicular bodies were absent, and vacuoles accumulated autophagic bodies in the lumen. (VII and VIII), Upon 30 min exposure to 10 µM ES9, the Golgi apparatus was even more affected and sometimes ER-Golgi hybrid compartments (white arrowhead) were formed in meristematic cells. (IX-XI), 1 µM carbonyl cyanide m-chlorophenyl hydrazine (CCCP) morphologically altered the Golgi and the *trans*-Golgi network, often by bending the stacks to form cup-shaped structures. CCCP also induced the accumulation of autophagic bodies in the vacuole, but did not form ER-Golgi hybrids and locally caused swelling of the ER cisternae. (XII), Quantification of the effects of DMSO, ES9, and CCCP. One-hundred Golgi and 100 vacuoles were analysed for each treatment; black, dark grey and light grey colours refer to altered, partly altered and normal compartments respectively. Scale bars, 200 nm.

Supplementary Table 1. Primers used in this study

Primer	Sequence 5´-3´
**Cloning**	
Nsi1-for	ATGCATCAACTTCGCGCTGCACAC
Sac1-rev	GAGCTCTCCTCCGCCTTGGTTCCCTC
SalI-for	TTTGTCGACTTTCCTTCTATTTGCGTTTGGCATTT TAACACTTG
SacI-rev	TTTGAGCTCTCCAACTTCTCTGTAACTGTGACCTG TTCACTC
CHC1fwd	GGGGACAAGTTTGTACAAAAAAGCAGGCTCCAC CATGGCGGCTGCTAACGCGCCC
CHC1rev-no stop	GGGGACCACTTTGTACAAGAAAGCTGGGTCGTA GCCGCCCATCGGTGGCATTC
**Site-directed mutagenesis**	
	5′-PO_4-TTGTCGTCTCTTTCCGTTAAAGCTCA-3′
	5′-PO_4-GGCGGGTCGGTTCTTCGATAAC-3′
